# The Chicken Frizzle Feather Is Due to an α-Keratin (*KRT75*) Mutation That Causes a Defective Rachis

**DOI:** 10.1371/journal.pgen.1002748

**Published:** 2012-07-19

**Authors:** Chen Siang Ng, Ping Wu, John Foley, Anne Foley, Merry-Lynn McDonald, Wen-Tau Juan, Chih-Jen Huang, Yu-Ting Lai, Wen-Sui Lo, Chih-Feng Chen, Suzanne M. Leal, Huanmin Zhang, Randall B. Widelitz, Pragna I. Patel, Wen-Hsiung Li, Cheng-Ming Chuong

**Affiliations:** 1 Biodiversity Research Center, Academia Sinica, Taipei, Taiwan; 2 Department of Pathology, Keck School of Medicine, University of Southern California, Los Angeles, California, United States of America; 3 Department of Anatomy and Cell Biology, Indiana University School of Medicine, Bloomington, Indiana, United States of America; 4 Department of Dermatology, Indiana University School of Medicine, Bloomington, Indiana, United States of America; 5 Department of Molecular and Human Genetics, Baylor College of Medicine, Houston, Texas, United States of America; 6 Institute of Physics, Academia Sinica, Taipei, Taiwan; 7 Taiwan International Graduate Program, Academia Sinica, Taipei, Taiwan; 8 Graduate Institute of Biotechnology, National Chung Hsing University, Taichung, Taiwan; 9 Department of Animal Sciences, National Chung Hsing University, Taichung, Taiwan; 10 Avian Disease and Oncology Laboratory, Agriculture Research Service, United States Department of Agriculture, East Lansing, Michigan, United States of America; 11 Institute for Genetic Medicine and Center for Craniofacial Molecular Biology, University of Southern California, Los Angeles, California, United States of America; 12 Department of Ecology and Evolution, University of Chicago, Chicago, Illinois, United States of America; 13 Research Center for Developmental Biology and Regenerative Medicine, National Taiwan University, Taipei, Taiwan; University of Colorado Health Sciences Center, United States of America

## Abstract

Feathers have complex forms and are an excellent model to study the development and evolution of morphologies. Existing chicken feather mutants are especially useful for identifying genetic determinants of feather formation. This study focused on the gene *F*, underlying the frizzle feather trait that has a characteristic curled feather rachis and barbs in domestic chickens. Our developmental biology studies identified defects in feather medulla formation, and physical studies revealed that the frizzle feather curls in a stepwise manner. The frizzle gene is transmitted in an autosomal incomplete dominant mode. A whole-genome linkage scan of five pedigrees with 2678 SNPs revealed association of the frizzle locus with a keratin gene-enriched region within the linkage group E22C19W28_E50C23. Sequence analyses of the keratin gene cluster identified a 69 bp in-frame deletion in a conserved region of *KRT75*, an α-keratin gene. Retroviral-mediated expression of the mutated *F* cDNA in the wild-type rectrix qualitatively changed the bending of the rachis with some features of frizzle feathers including irregular kinks, severe bending near their distal ends, and substantially higher variations among samples in comparison to normal feathers. These results confirmed *KRT75* as the *F* gene. This study demonstrates the potential of our approach for identifying genetic determinants of feather forms.

## Author Summary

With the availability of a sequenced chicken genome, the reservoir of variant plumage genes found in domestic chickens can provide insight into the molecular mechanisms underlying the diversity of feather forms. In this paper, we identify the molecular basis of the distinctive frizzle (*F*) feather phenotype that is caused by a single autosomal incomplete dominant gene in which heterozygous individuals show less severe phenotypes than homozygous individuals. Feathers in frizzle chickens curve backward. We used computer-assisted analysis to establish that the rachis of the frizzle feather was irregularly kinked and more severely bent than normal. Moreover, microscopic evaluation of regenerating feathers found reduced proliferating cells that give rise to the frizzle rachis. Analysis of a pedigree of frizzle chickens showed that the phenotype is linked to two single-nucleotide polymorphisms in a cluster of keratin genes within the linkage group E22C19W28_E50C23. Sequencing of the gene cluster identified a 69-base pair in-frame deletion of the protein coding sequence of the α-keratin-75 gene. Forced expression of the mutated gene in normal chickens produced a twisted rachis. Although chicken feathers are primarily composed of beta-keratins, our findings indicate that alpha-keratins have an important role in establishing the structure of feathers.

## Introduction

Birds have evolved many unique and interesting features, allowing them to adapt and radiate into various ecological niches. They display a great degree of diversity in feathers and other body parts. Domesticated birds exhibit an even greater diversity in phenotypes than their wild ancestors, thus providing an excellent opportunity to explore the genetic basis underlying variation in morphology, physiology, and behavior.

As Darwin noted, the domestic chicken displays a remarkable level of phenotypic diversity [1] and it is the most phenotypically variable bird, especially in terms of feather form [2]. However, the genetic and developmental basis of this diversity is unclear. Understanding the genetic basis of plumage variability in the chicken would provide insight into how evolutionary diversification in morphological traits could occur rapidly during adaptive radiations or under strong sexual selection.

The development of a feather has to be coordinated by an enormous number of molecular and cellular machineries [3]–[17]. The feather is the most complex keratinized structure of the vertebrate integument and has vital importance for physiological and functional requirements. The complex organization of feathers allows for a variety of potential morphological changes to occur. Modifications of the feather include deterrence of feather development, changes in feather structure, inhibition of feather molting, and alterations of feather growth rates [18].

**Figure 1:**
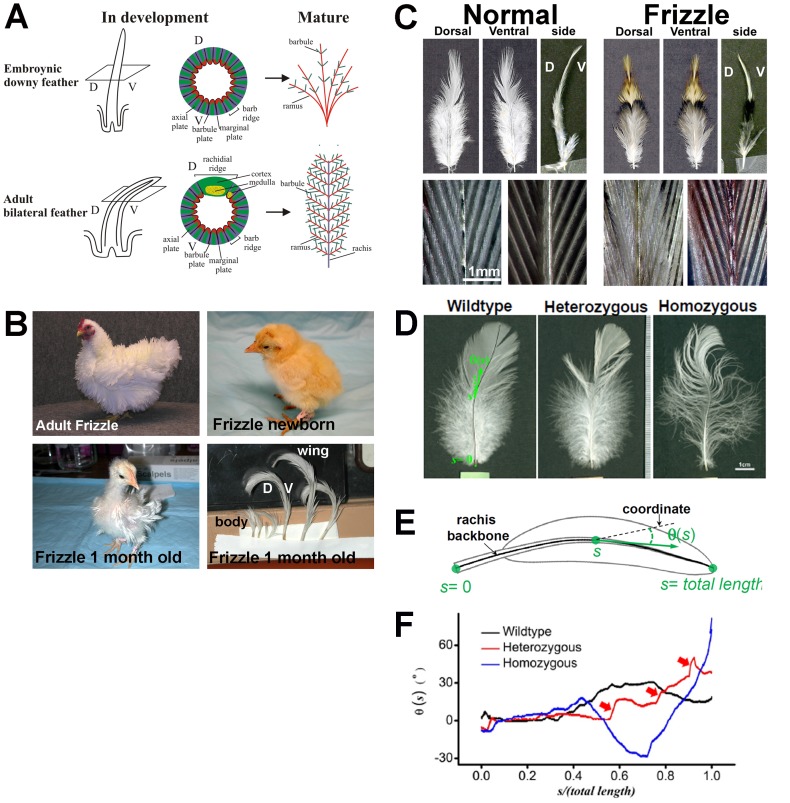
The frizzle chicken phenotype. (A) Diagram of normal developing and mature embryonic and adult chicken feathers. (B) Adult, hatchling and 1-month-old frizzle chickens. Adult homozygous frizzle chicken feathers curve away from the body. This is a frizzle in White Plymouth Rock Bantam, it is not exactly the chicken we use but illustrate the phenotype. Note that the downy feathers appear normal in newborn frizzle chicks; however, by the second generation, the feathers in a one month old chick start to show a clear frizzle phenotype. (C) Comparison of body feathers from normal white leghorns and frizzle chickens. Upper panel: dorsal view, ventral view and side view. Lower panel: dorsal view (left) and ventral view (right) of branching in the pennaceous vane; D, dorsal; V, ventral. (D) Comparison of the wildtype, heterozygous and homozygous frizzle feathers. The wildtype feather image is overlaid by the computer-determined backbones of its rachis. (E) Function θ(*s*) describing the bending of their rachis, plotted on the length-normalized coordinate. The functions are shifted by arbitrary offsets for clarity. (F) Comparison of the feathers as shown by the qualitative change of the curves of θ(*s*). Red arrows highlight the kinked structures along the heterozygous feather.

The structure of feathers includes the rachis (feather backbone), ramus (branches) and barbules (branches off the ramus, which enable them to form an organ capable of moving air to provide flight) (Figure 1A). In the chicken, embryonic downy feathers are radially symmetric and fluffy and have a very short rachis or none at all. The branches in downy feathers only include the ramus and barbules (Figure 1A). Most adult chicken feathers are bilaterally symmetric and include a rachis, ramus, and barbules (Figure 1A). The rachis and ramus are composed of two layers: the outer, thin cortex (composed of solidly compacted squamous cells) and the inner, thick medulla (composed of empty polyhedral pith cells) [19]. Various feather types are essential characteristics of domestic chicken breeds. Although the molecular and cellular basis of feather development has been well characterized [3]–[6], little is known about the genes influencing feather growth, pigment pattern, length, distribution, and structure.

The existence of a reference genome sequence has placed the chicken as an important model organism for understanding genome evolution, population genetics, and the genetic basis of phenotypic traits [20]–[33]. Owing to the close relationships within Class *Aves*, the molecular and genetic understanding of phenotypic variations discovered in chickens are likely to be applicable to wild bird species. Therefore, chicken genetic and genomic studies can provide information for studying development and evolution of avian species [2], [34]–[42].

Frizzle feathers have been described in domesticated birds and established as varietal characteristics in domestic chickens [18], [43]. The contour feathers of the frizzle chicken all curl outward and upward. Due to an altered feather rachis structure and morphology, they cannot lie flat against the body. Usually, rectrices and remiges are less affected by the mutation but have an irregular appearance. Other prominent modifications, such as thickening of the barbs and barbules, alteration of the hooklets and other structural abnormalities have also been observed [44]. The frizzle mutation has been reported to occur in a single autosomal gene denoted *F* that shows incomplete dominant inheritance [44], [45].

In order to dissect the genetic mechanism underlying frizzle feathers, we conducted a whole genome linkage scan and mapped the causative genetic mutation to the linkage group E22C19W28_E50C23. By analyzing the candidate genes in the associated interval, we found that the *F* mutation is caused by a deletion in a conserved region of an α-keratin. The causative effect of the KRT75-MT was confirmed by a retrovirus-mediated misexpression of the wild-type or mutated K75 protein in the feather follicle during regeneration in chickens with normal plumage.

Interestingly, mutations in *KRT75* have also been identified in mammals, causing structural abnormalities in hair in humans [46], [47] and mice [48]. This implies a fundamental role for K75 in building the architecture of skin appendages.

## Results

### Physical features of the frizzle chicken

The adult frizzle chicken shows a distinct disorientation of feathers (Figure 1B). Upon hatching, the first-generation radially symmetric feathers of frizzle chicks do not show curves (Figure 1B). The frizzle phenotype starts to appear when the first-generation feathers are replaced with second-generation bilateral feathers which have a rachis. At this stage, both body and wing flight feathers twist toward a dorsal orientation.

Normally, feathers are bent along the dorsal to ventral orientation. However, in frizzle chickens, the feathers are bent along the ventral to dorsal orientation (Figure 1C). The pennaceous vane on both dorsal and ventral sides of frizzle feathers show normal branching when compared to white leghorn controls (Figure 1C). Their rachis backbones are determined by our computer-aided analyses (Figure 1D). The definitions of “*s*” and “θ” are shown in the schematic drawing of a feather in Figure 1E. We define the backbone of the rachis by a curve at equal distance to the two edges of the rachis, because rachis has a width. The accumulated distance from the most proximal end of the rachis along the backbone is defined as “*s*”. We then, arbitrarily draw a straight line as the coordinate, and define the angle between the local tangent line (the best straight-line approximation to the rachis backbone at that point) and the straight line as “θ”. Thus the bending of the rachis is represented by the function θ(*s*), which quantitatively reflect the changing curvature of different feathers (Figure 1E). Features of θ(*s*) quantitatively reflect some subtle differences beyond a visual inspection of the images. For example, the heterozygous chicken feather shows not only kinks along its length but also curves over the entire rachis in comparison to the homozygous chicken whose feathers have dramatically increased curvature and less kinking (Figure 1F). These features may be correlated to the growth of the feather in response to the expression of a different allele.

### The cellular basis of the frizzle phenotype

**Figure 2:**
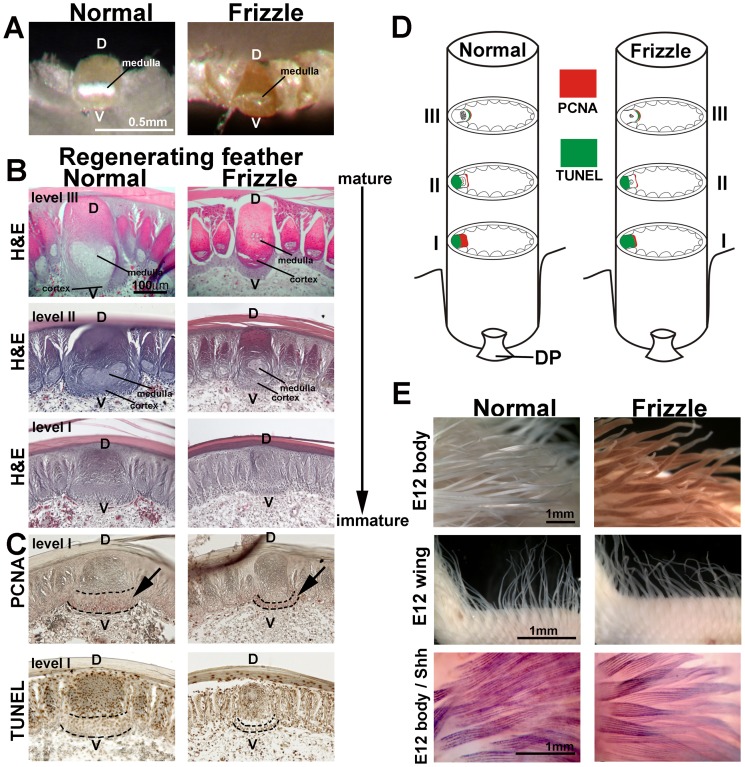
Histological sections of developing normal and frizzle feather filaments. (A) Top view of a cross section through the rachis in a pennaceous vane. (B) Upper three panels: H&E staining of sections at different levels from mature (feather tip) to immature (feather base). (C) PCNA (upper panel) and TUNEL (lower panel) staining of the sections adjacent to the immature section. (D) Diagram summary of PCNA and TUNEL staining at different levels of the rachis. (E) Comparison of E12 body feathers (upper panel) and wing feathers (middle panel) between normal and frizzle embryos. Lower panel, whole mount *in situ* hybridization of *SHH*. D, dorsal; DP, dermal papilla, V, ventral.

Feather sections from the mature, top region show that the rachis of frizzle feathers has a smaller medulla compared to the normal leghorn controls (Figure 2A). The medulla is localized in the inner, ventral region of the rachis and is composed of empty polyhedral pith cells [19]. These observations suggest that the frizzle phenotype is caused by a defect in the ventral part of the rachis.

We examined rachidial morphogenesis at different regeneration time points by plucking body feathers and allowing them to regenerate for 10 days or 30 days. After regeneration, feather follicles were dissected and paraffin sections were prepared. Three different levels of cross-section of a 30-day regenerated sample, from mature (Figure 2D and S1A, level III) to immature (Figure 2D and Figure S1A, level I), were studied by H&E staining (Figure 2B). These results indicate that a clear defect in the ventral region of the rachis may be responsible for the altered medulla formation. PCNA (Proliferating Cell Nuclear Antigen) staining showed that the cell proliferating zone in the frizzle rachis in level I is much narrower than that of the controls (Figure 2C, upper panel). The TUNEL staining showed there is no cell death in the PCNA positive cell proliferating zone (Figure 2C, lower panel). Detailed PCNA and TUNEL staining (Figure S1B–S1E), summarized in Figure 2D and Figure S1F, show there are no differences in cell proliferation and programmed cell death between normal and frizzle feathers at level II and level III. Our cell proliferation data suggests that the cell proliferation zone at an immature level (level I) of the frizzle rachis is narrow compared to that found in a normal rachis, perhaps contributing to smaller medulla formation in the frizzle rachis.

Finally, we compared the feathers of homozygous frizzle chickens with white leghorn controls at embryonic day 12. The base of the feather filament appeared normal. However, the tip of each frizzle feather filament appears to be randomly twisted in both the body and wing feathers (Figure 2E, upper two panels). To examine possible differences between frizzle and normal feather branching morphogenesis, we used whole mount *in situ* hybridization with a probe targeting *SHH* which is expressed in marginal plate cell [49]. The frizzle feathers showed the same expression of *SHH* as controls (Figure 2E, lower panel). This suggests that embryonic frizzle feather branching occurred normally even though the tip of frizzle feathers were randomly twisted.

### Linkage analysis maps the frizzle trait to the linkage group E22C19W28_E50C23

**Figure 3:**
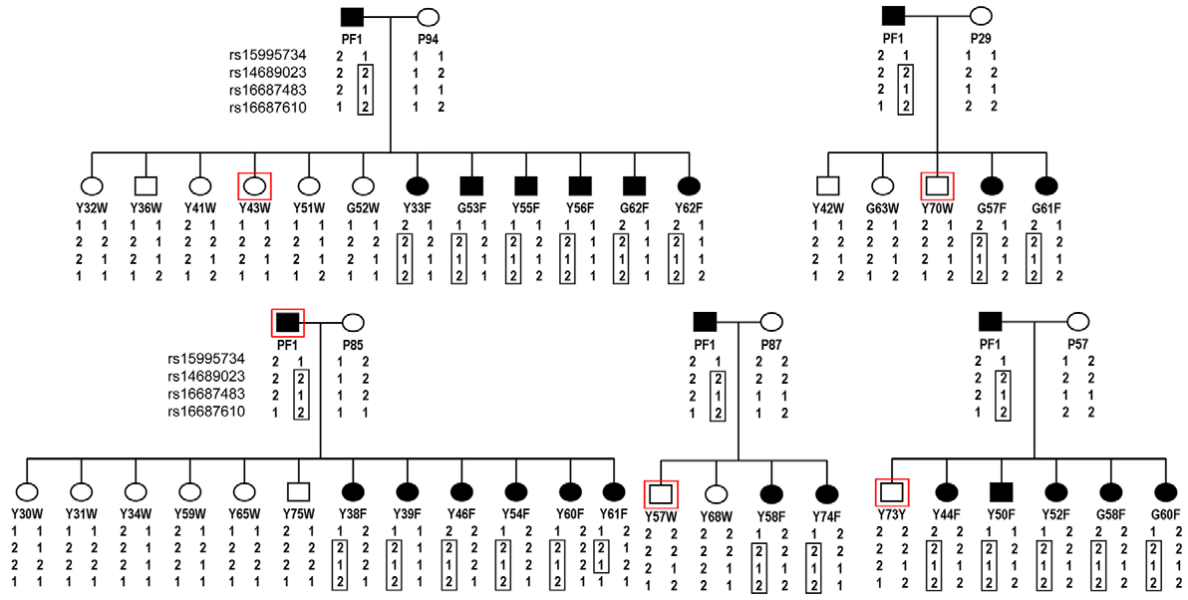
Pedigrees of frizzle chickens used for mapping of the frizzle locus by linkage analysis. A single frizzle rooster, PF1 was bred to five different wild-type hens. DNA was extracted from the offspring from these matings, the five hens and the single rooster parent and used for the genome scan. The SNPs, rs16687483 and rs16687610 within the linkage group E22C19W28_E50C23 yielded LOD scores of 7.34 and 6.5, respectively. Haplotypes for these latter SNPs and two others from the same chromosome that were represented in the SNP panel used for genotyping are shown. The haplotype of SNPs segregating with the frizzle phenotype is delineated by the boxed genotypes.

In order to locate the gene underlying the frizzle trait, a genome scan was conducted on progeny of crosses between the same heterozygous frizzle rooster, PF1, and five different wild-type feathered hens. A total of 2678 SNPs were genotyped in 45 birds and linkage analysis of the genotyping data identified two SNPs, rs16687483 and rs16687610 within the linkage group E22C19W28_E50C23 that yielded a LOD score of 7.34 and 6.5, respectively. Haplotype sharing of SNPs between family members identified a shared haplotype extending from rs14689023 to rs16687610 in 21 of 22 frizzle birds. A possible recombination event in the region between rs16687483 and rs16687610 was evident in the frizzle female Y61F (Figure 3).

### Identification of a mutation in *KRT75* in the frizzle chicken

A cluster of keratin genes was found within the genomic interval to which the frizzle locus was mapped by the above analysis. Mutations in keratins are obvious candidates for altered feather phenotypes [50], [51]. Keratins purified from the frizzle feather showed a slightly altered amino acid content, produced distinct X-ray diffraction patterns, and exhibited quantitative banding changes on SDS-PAGE gels [52], [53].

**Figure 4:**
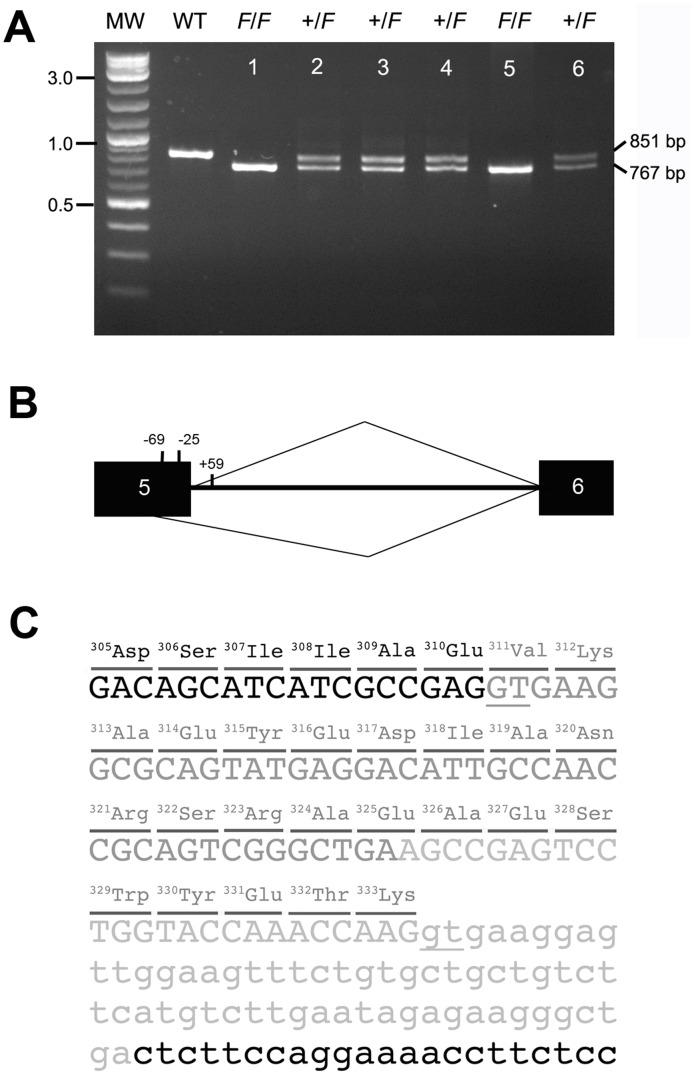
The *F* allele of *KRT75* contains a deletion. (A) PCR products amplified from genomic DNA of phenotypically normal (WT), heterozygous (+/*F*, lanes 2–4, 6), and homozygous (*F*/*F*, lanes 1, 5) frizzle chickens. The normal band is 851 bp and the mutant band is 767 bp. The size of the molecular marker (MW) is showed in kb. (B) Diagram of the chicken *KRT75* and the cryptic splice site activated by the deletion mutation that covers positions −24 of exon 5 to +59 of intron 5. Black boxes represent exon sequences; intron 5 is designated by a line. The caret designating use of the cryptic site (position −69) is shown below, and the caret designating use of the authentic site is shown above the diagram of the pre-mRNA. (C) Partial sequence of the *F* allele. The 84-bp deletion in genomic DNA is shown in light gray letters. The additional deletion in exon 5 created by a cryptic splice site is shown in dark gray letters. The deletion in genomic DNA and use of the cryptic splice site together result in a deletion of 23-amino acids (position 311–333) in the K75 protein. Parts of exon 5 and intron 5 are shown in capital and small letters, respectively. The underlines show the authentic and cryptic mRNA splicing sites.

To identify possible causative variants, we PCR-amplified and sequenced partial gene regions of the 14 keratin candidate genes (Table S1) and found only one significant variation in a coding sequence (GenBank accession number JQ013796), namely, a deletion covering the junction of exon 5 and intron 5 in the *KRT75* gene (chrE22C19W28_E50C23:658,389–658,472) (Figure S2, Table S2). This deletion mutation showed complete segregation with the frizzle phenotype in all the frizzle offspring within the F1 generation of the experimental crosses (Figure S3 and Figure S4). Frizzle chickens sampled from different populations in Taiwan with the distinctive homozygous and heterozygous feather phenotypes demonstrated two mutant alleles and a single mutant allele, respectively (Figure 4A). The deletion was not observed in other breeds of normal chickens. Other variants discovered by sequencing genomic DNA from the frizzle chicken were also found in non-frizzle chickens except for one nonsynonymous SNP (Table S2). The effects of variants in other genes were not subjected to functional studies.

We isolated RNA from the feather follicles 2-weeks after plucking of normal and *F*/*F* chickens and surveyed the expression of *KRT75* in the feather follicles. We confirmed that *KRT75* is expressed in feather follicles of both normal and *F*/*F* chickens (Figure S5). Sequence analysis of the coding sequence of *KRT75* cDNA showed that the loss of the authentic splice site at the exon5/intron 5 junction activates a ‘cryptic’ splice site in exon 5 (Figure 4B), resulting in a 69-bp in-frame deletion within the coding region (CDS positions 934–1,002). The cryptic splicing site in exon 5 contains 6-bp (5′-GTGAAG-3′) that resembled those at the authentic splice site. The mutated K75 thus contains a deletion of 23-amino acids within a conserved region (Figure 4B and Figure S6). The deletion covers the entire part of link L2 and some parts of the coiled-coil segments of 2A and 2B in K75 (Figure S7) [54]. The length of link L2 is highly conserved in all keratin proteins and required for changing the azimuth of the coiled-coil over a short distance axially to reorient the apolar residues in coiled-coil segment 2A appropriately in terms of energetic stability [55]. Therefore, the loss of link L2 might significantly disrupt the structure over the coiled-coil segments 2A and 2B, potentially preventing the proper dimerization of keratin, consistent with a dominant-negative mode of action.

### Expression of *KRT75* in embryonic and adult feathers

**Figure 5:**
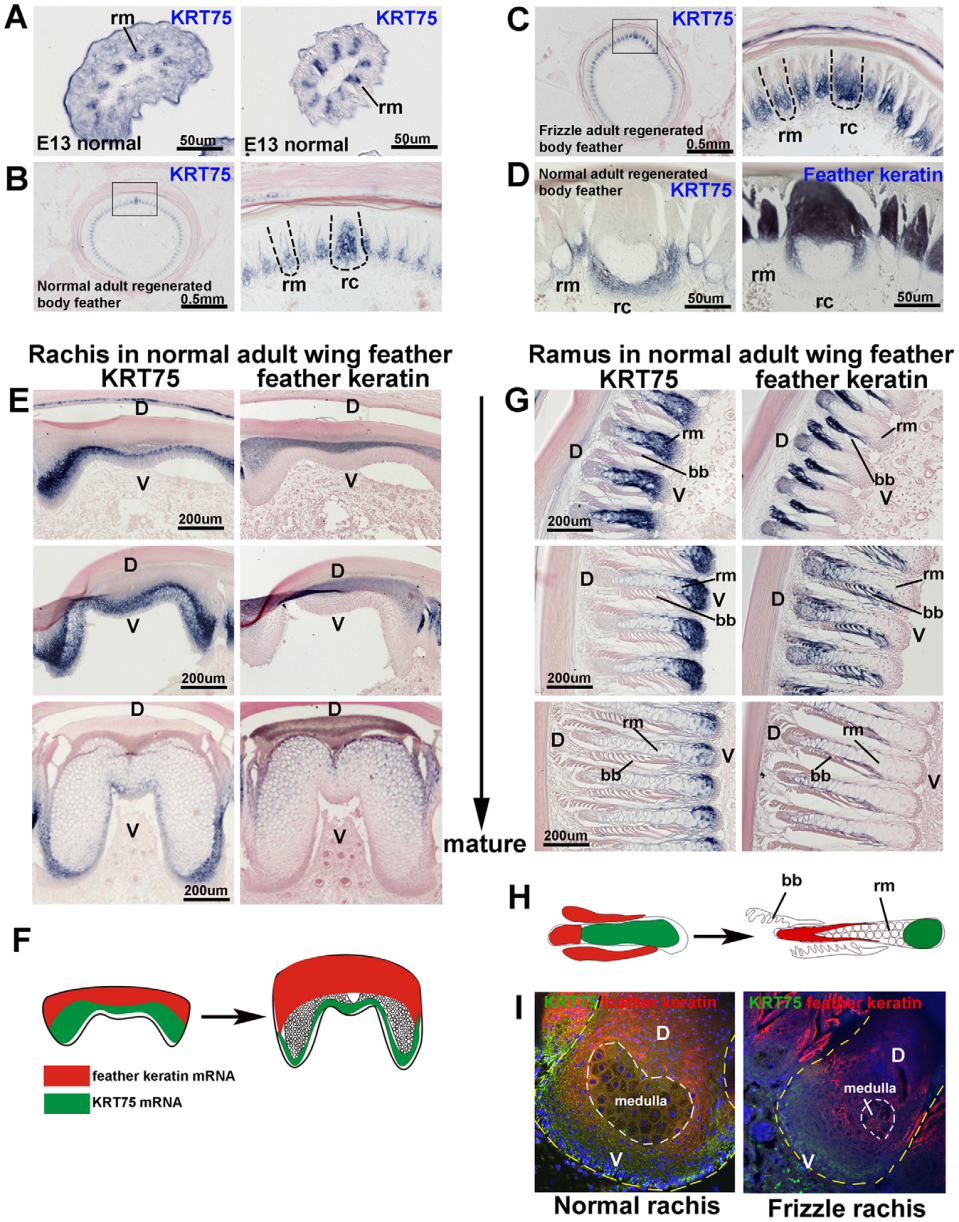
*In situ* hybridization of *KRT75* in embryonic and adult feather filaments. (A) Cross section of E13 feather filaments. *KRT75* is expressed in the region that is destined to become the ramus. Note that embryonic downy feathers do not have a rachis. (B) *KRT75* is expressed in the rachis and ramus in adult normal regenerating feathers. (C) *KRT75* is expressed in the rachis and ramus in adult frizzle regenerating feathers. (D) A nearly mature rachis expresses *KRT75* in the ventral part of the rachis. In comparison, feather keratin is expressed in the dorsal part of the rachis. (E) Sections of a rachis from adult wing feathers at different levels of maturity from the feather base (immature) to the feather tip (mature). *KRT75* is expressed in the ventral part of the rachis. In comparison, feather keratin is expressed in the distal part of the rachis. (F) Schematic drawing which summarizes the expression pattern of *KRT75* and feather keratin adult feather rachis. (G) Barb ridge of adult wing feathers at different levels of maturity. *KRT75* is expressed in the ventral part of the ramus. In comparison, feather keratin is expressed in the distal part of the rachis. (H) Schematic drawing which summarizes the expression pattern of *KRT75* and feather keratin in adult feather ramus. (I) Double immunostaining for K75 (green) and feather keratin (red) in the rachis of normal and frizzle feathers. The yellow dotted line outlines the rachis and the white dotted line indicates the medulla. D, dorsal; V, ventral; bb, barbule; rc, rachis; rm, ramus.

To locate the *KRT75* transcripts in embryonic and adult feathers, we generated a *KRT75* full-length antisense RNA probe. Section *in situ* hybridization showed that *KRT75* is expressed in barb ridges but restricted to the region destined to become the ramus, at embryonic day 13 (E13) (Figure 5A). In the normal regenerating adult feather, we found that *KRT75* was expressed in both the rachis and the ramus (Figure 5B). To ensure the specificity of our *KRT75* probe, we made probes from the 3′untranslated region (UTR) which show the same expression pattern as our probe to the coding region (data not shown). The regenerating frizzle feathers show the same pattern of *KRT75* expression as those in normal controls (Figure 5C, compared to 5B), suggesting that *KRT75* mRNA is expressed in the normal regions in the frizzle mutant chicken feathers and the phenotype must result from a dysfunction of the protein.

We then compared the expression of *KRT75* and feather keratin at mature regions of the wildtype body feather (Figure 5D). We found that *KRT75* and feather keratin were co-expressed in the rachis and ramus. *KRT75* was expressed in the ventral part, whereas feather keratin was expressed in the dorsal part of the rachis and ramus.

To study the expression pattern differences between *KRT75* and feather keratin, we examined the expression of these two keratins in the rachis and ramus from the immature to the mature stage of normal wing feathers (Figure 5E–5H). *KRT75* is expressed in the ventral part but feather keratin is expressed in the dorsal part of the immature rachis. Ventral regions of the feather formed a medulla that expressed *KRT75*, whereas feather keratin was expressed in the dorsal region (Figure 5E, 5F). In the ramus, *KRT75* and feather keratin were expressed in a similar pattern as in the rachis (Figure 5G, 5H). In summary, in both the rachis and ramus, *KRT75* and feather keratin were expressed in a complementary pattern (Figure 5F, 5H). K75 was present in the medulla but feather keratin was not. These data confirm that although body and wing feathers have differences in symmetry and size, they show similar expression patterns of *KRT75*.

To further examine the alteration of K75 protein expression in the frizzle rachis, we performed double immunostaining using antibodies to both K75 and feather keratin. Figure 5I shows staining of a section at level II, adjacent to the section shown in Figure 2B. In the normal rachis, feather keratin protein (red) is expressed in the dorsal part and in regions surrounding the medulla, while K75 protein (green) is expressed in the ventral rachis as well as at lower density in the medulla. The protein expression pattern is the same as the mRNA expression pattern (Figure 5D). In the frizzle rachis, K75 protein is only expressed in the narrower ventral region but the feather keratin domain expanded to cover the medulla which is reduced in size. The perturbed keratin organization in the frizzled rachis suggests that the frizzled phenotype may be caused by the *KRT75* mutation.

### Effects of misexpressing KRT75-WT and KRT75-MT on embryonic feather development

**Figure 6:**
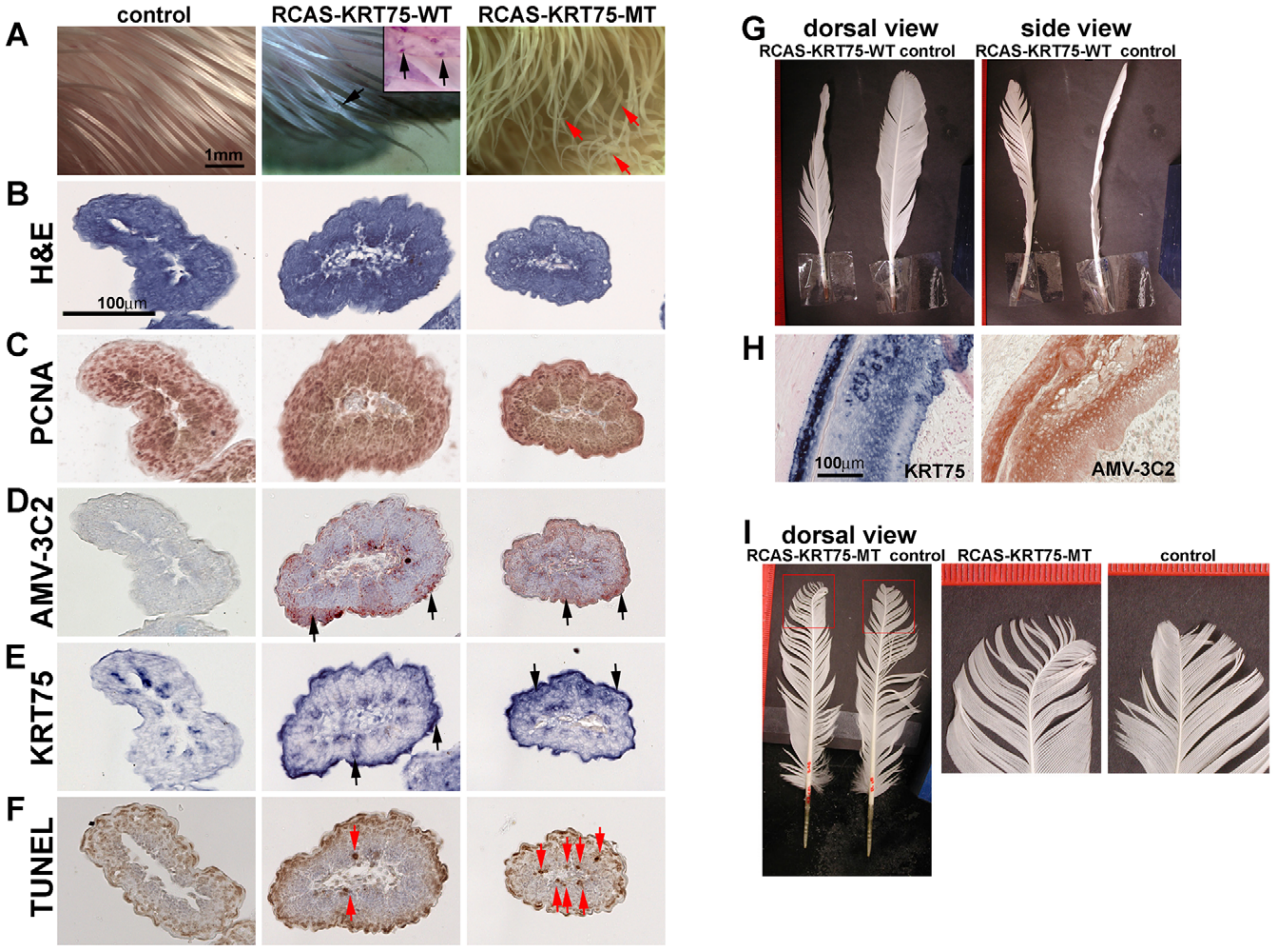
Misexpression of KRT75-WT and KRT75-MT in embryonic and adult feathers. (A) Misexpression of KRT75-WT and KRT75-MT in embryonic feathers. Left panel, control; middle panel, KRT75-WT; right panel, KRT75-MT. KRT75-WT misexpression produced some keratin deposition within the feather filaments (see inset in middle panel, black arrows). KRT75-MT misexpression generated feathers with curved tips (red arrows), which mimics embryonic feathers in the frizzle chicken. (B–F) Characterization of embryonic feathers by H&E, PCNA, AMV-3C2 and TUNEL staining at level III. Left panel, control; middle panel, KRT75-WT; right panel, KRT75-MT. We observed cross sections along the proximal-distal axis and only present the distal part here: H&E staining (B); PCNA staining (C). AMV-3C2 staining of adjacent sections showing the RCAS virus in the infected feather filaments (D, black arrows). The *in situ* hybridization probe to *KRT75* stains both endogenous and exogenous KRT75 (E, black arrows). TUNEL staining. KRT75-MT misexpression increases apoptosis significantly above control and RCAS-KRT75-WT specimens (F, red arrows). (G) Misexpression of KRT75-WT in adult feather follicles. KRT75-WT generates twisted feathers. (H) Section *in situ* hybridization shows the expression of ectopic *KRT75* and AMV-3C2 staining in RCAS-KRT75-WT transduced feather follicles. (I) Misexpression of KRT75-MT in adult feather follicles. KRT75-MT generated feathers with curved tips.

To test the function of *KRT75* in feather development, we constructed RCAS-KRT75-WT and RCAS-KRT75-MT viruses to misexpress the normal and mutant forms in embryonic and adult chickens. RCAS-KRT75-WT virus did not produce feather malformations in chicken embryos; however, some keratin-like depositions were found (N = 10/10) (compare middle and left panels in Figure 6A). Whole mount *in situ* hybridization of *KRT75* confirmed the ectopic expression of *KRT75* in the feather filaments (insert in Figure 6A, middle panel). In comparison, misexpression of KRT75-MT generated feathers with curved tips, mimicking the frizzle phenotype (N = 8/20) (Figure 6A, right panel).

We further characterized feather phenotypes produced as a result of *KRT75* misexpression by H&E staining, PCNA staining, AMV-3C2 staining (for RCAS virus detection), *KRT75* section *in situ* hybridization and TUNEL assay (for detection of apoptosis) in serial paraffin sections at different levels of the filament (from proximal - level I, to distal - level IV, as shown in Figure S8). Figure 6B–6F shows sections at level III, which are close to the feather filament tip. We did not detect significant alterations based on H&E (Figure 6B) and PCNA staining (Figure 6C) among the treated samples and controls. The treated samples displayed strong AMV-3C2 staining (Figure 6D, arrows) and ectopic *KRT75* expression (Figure 6E, arrows). The detailed H&E and TUNEL staining at different levels are shown in Figure S8B–S8D′. In normal development, programmed cell death appeared in the peripheral epidermis at level I, II and III but eventually apoptosis occurred in all distal tip cells (level IV) (Figure S8B′). Cell death was detected infrequently in the proximal to middle region of the epidermis shown in Figure S8B′ (red arrow). However, both treated samples induced ectopic cell apoptosis but KRT75-MT misexpression induced significantly increased TUNEL positive cells (Figure 6F, Figure S8C′ and S8D′). We conclude that the misexpressed mutant form of *KRT75* induces significant ectopic cell apoptosis, which may be responsible for the randomly curved feather morphology in the RCAS-KRT75-MT infected feathers.

### Effects of misexpressing KRT75-WT and KRT75-MT on adult feather morphology

To verify that the adult frizzle phenotype is due to the identified *KRT75* mutant, we misexpressed KRT75-WT or KRT75-MT by injecting the RCAS virus into adult feather follicles after plucking. Misexpressing KRT75-WT produced twisted feathers (N = 5/12) (Figure 6G). The control feathers involving only plucking or injecting RCAS-GFP did not show the twisted phenotype (N = 0/20). Cross sections of the twisted feathers showed the asymmetrical distribution of ectopically expressed *KRT75* in the ramogenic zone of the feather follicle (Figure 6H). Misexpression of the mutant form of *KRT75* produced the curved feathers but the curvature only existed at the tip of the feather (N = 6/10) (Figure 6I). Control feathers on the right wing did not show any unusual curvature (N = 0/10) (Figure 6I). Since misexpressing the mutant form of *KRT75* in a normal feather follicle that contains numerous normal *KRT75* transcripts only affects the distal feather tip, we presume that its effect may be masked by high levels of endogenous wild type transcripts and limited to the softest part of the rachis at the tip.

**Figure 7:**
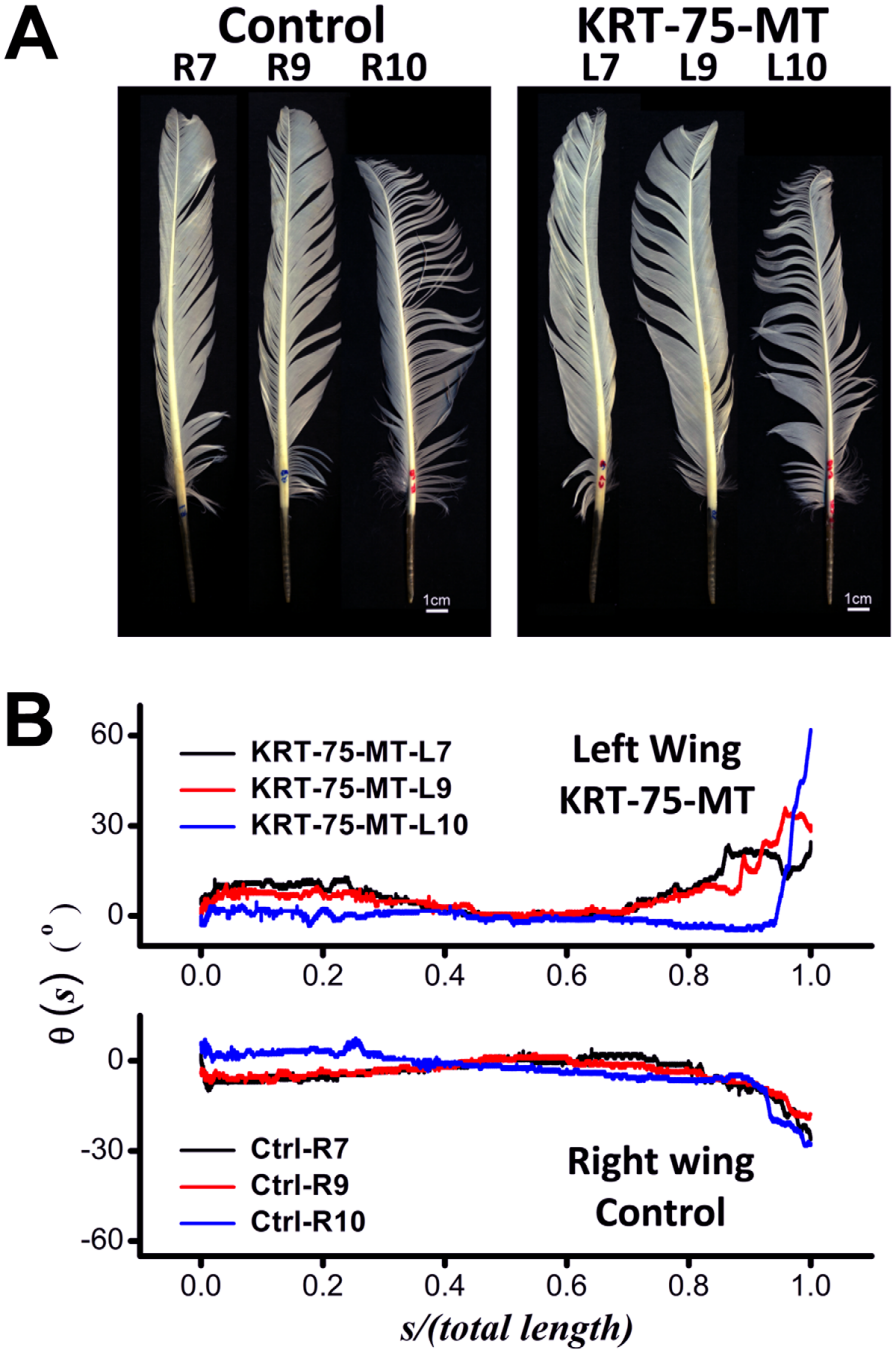
Contrast between feathers that have misexpressed GFP (Control, right wing) and KRT75-MT (left wing). (A) Images of flight feathers from both sides of the chicken (the R*n* and L*n* denote the *n* ^th^ feather taken from the right and left wing, respectively). (B) Effects of the viral misexpression, as shown by the qualitative change of the curves of θ(*s*) from the controls. Without the gene mutation, the trend of curves θ(*s*) obtained from opposite sides of a normal chicken were expected to exhibit mirror symmetry, which is obviously abrogated in this case.

Images of flight feathers sampled from two wings of the same chicken in our experiments are shown in Figure 7A. Even though visual inspection of images of the control and KRT75-MT transfected feathers only reveal subtle differences, computer-aided analyses showed that ectopic expression of mutant K75 substantially changed the way the feathers bend along their rachis. Under normal circumstances, the natural bending of feathers from either side of a wildtype chicken would be expected to display reflective or mirror image symmetry to that of the opposite wing (Figure S9). Instead of the wild-type gentle inward bend, the end of the infected feather was twisted abruptly away from the body (Figure 7B), as a consequence of the viral KRT75-MT misexpression during the feather growth of the left wing. While θ(*s*) of three control feathers generally converge on the length-normalized coordinate, these curves of θ(*s*) determined from the KRT75-MT transfected feathers are rather diverse. They exhibit anomalous bending and kinky structures that are qualitatively different from those of the controls. Our analyses also revealed that the over-expression of KRT75-WT resulted in twisted feathers and increased the curvature of the feather in a smooth manner (Figure S10), suggesting that excessive K75 may affect the physical properties of the feather.

### Expression of KRT75-MT disrupts the intermediate filament cytoskeleton in mammalian cells

Ectopic mouse K75 (K6hf) was reported to co-localize with K8, K17 and K18 in cultured PtK2 rat kangaroo kidney epithelial cells [56]. To explore the role of KRT75-MT in disrupting the intermediate filament structure in a dominant fashion, we transfected PtK2 cells with RCAS expressing either KRT75-WT or KRT75-MT. K18 (red) is present in the cytosplasm in a network configuration and K75 (green) is weakly positive in control PtK2 cells (Figure S8E). After transfection with wild type KRT 75, KRT 75 is expressed strongly. The keratin network is still maintained (Figure. S8F). When the mutant K75 form is expressed, both K18 and K75 accumulate around the nucleus (Figure S8G, white arrows). This is similar to what was found for mouse K75 [48]. Our data suggests that avian KRT75-MT can act in a dominant negative fashion to disrupt the keratin filament network.

## Discussion

Compared to traditional model organisms, domesticated animals have a number of advantages. Selection is not based on a need for survival in nature, but rather upon human preferences due to economic or aesthetic values. This allows selection for extreme phenotypes that may not survive in the wild. Large populations and greater longevity mean that mutations of biologically important traits have a greater chance of appearing and of being maintained, giving us an excellent opportunity to identify novel functions for specific genes. In the case of chickens, the availability of breeds selected for economic value or fancy feather variants, and the progress in chicken genomics have provided a prime opportunity to study the genetic basis of feather morphogenesis [57]. We have taken advantage of the availability of genome-wide SNPs and the chicken genomic sequence to identify the molecular basis of frizzle feathers. Our work reveals the important role of α-keratin in the development and differentiation of feather structures.

### Feather keratins

*KRT75* is a member of the type II epithelial α-keratin gene family [58]–[60]. The feather mainly consists of two types of keratin proteins: α- and β-keratins. An obligate heteropolymer is formed by two types of α-keratin, an acidic type and a basic/neutral type, and culminates into the 8–10 nm-thick intermediate filament [61], [62]. The polymerization partner of K75 is unclear but it may be K17 in mammals [48]. In contrast to α-keratin, a fibrous protein rich in alpha helices, β-keratin is rich in stacked β-pleated sheets. β-keratins are only found in reptiles and birds, whereas α-keratins exist in all vertebrates [50].

K75 is not a hard feather keratin per se. Although the feather mainly consists of feather-specific β-keratins, cellular and biochemical studies have shown that α-keratin plays an important role in the early formation of rachides, barbs, and barbules [51]. The molecular mechanisms for accumulating α-keratin in down feathers and regenerating feathers are still largely unknown. It has been proposed that during the development of regenerating feathers, the α-keratin in the initial tonofilaments of sheath cells is replaced by feather-specific β-keratin [51], [63], [64]. Ultrastructural studies indicate that bundles of keratin filaments of 8–12 nm in diameter (α-pattern) are initially formed in differentiating barb/barbule cells and later replaced by 3–4 nm-thick filaments (β-pattern) [51], [65]. Our studies however, revealed that the α-keratin and β-keratin actually accumulated in different parts of the rachis and ramus. We found that *KRT75* is expressed in the ventral part that is destined to become the medulla, whereas β-keratin is expressed in the dorsal part of the rachis and ramus that is destined to become the cortex.

Biochemical studies have indicated that feather α-keratins are mainly acidic, while basic α-keratins are thought to be rare in feathers [51]. Cytokeratins have been proposed to have a role in the formation of an initial and temporary scaffold for the deposition of immense quantities of compact feather keratins [51]. The identification of *KRT75*, which encodes a type II cytokeratin (basic), as a major determinant of normal feather structure suggests that basic α-keratins are also critical for feather formation. In the cytoplasm of rachis sheath cells, a higher quantity of α-keratin bundles is initially deposited. This observation may explain why the rachis is more severely affected than the barb and barbule in *KRT75* mutant chickens.

During feather filament development, keratinocytes eventually die, either leaving space or leaving a keratinized structure. Before those events occur, localized proliferation and apoptosis of keratinocytes either add or remove cells in different places, thus shaping the feather, including the rachis [66].

Our data show that apoptosis is expanded in frizzle compared to control adult feathers in the immature feather region. In contrast, the proliferation zone is decreased in frizzle chicken feathers compared to controls. Our functional studies on embryonic chicken feathers show that apoptosis is increased within the inner epithelium of embryonic feathers expressing ectopic *KRT75* compared to controls and is increased even further in embryonic feathers expressing the mutant form of *KRT75*. Currently, we do not know whether the mechanism is through a classic mechanical role or through an alternate pathway as was seen for *KRT17* in mammalian hair follicles [67].

Our results show that *KRT75* unquestionably plays a significant role in the normal development of feathers. However, the cellular mechanisms underlying the frizzle phenotype have not been specifically probed because the role of α-keratin in feather development is largely unexplored. The mutation could potentially affect feather formation in many ways such as altering the mechanical properties of the feather, weakening the initiation of keratin formation, causing abnormal scaffolding for feather keratin deposition, impairing α- and β-pattern replacement, or perturbing β-keratin polymerization. Our findings support the importance of α-keratin in feather formation. Our results also demonstrate the power of using mutants of domestic chickens as a genetic model to unravel biological functions that are difficult to reveal by traditional biochemical and cellular studies.

### Effects of the chicken frizzle mutation

The action of the *F* gene is localized in the feather follicle and is not a consequence of a metabolic disorder [68]. However, the *F* gene may also have other pleiotropic effects that cause physiological abnormalities. Frizzle plumage may cause the acceleration of basal metabolism due to the loss of body heat, leading to alterations in organ size (e.g., enlargement of the heart, spleen, gizzard, and alimentary canal as well as lack of hypodermal fat deposits) and numerous physiological anomalies (e.g., higher food intake, oxygen consumption, heart rate, volume of circulating blood as well as delayed sexual maturity or decreased fertility) [18], [68]–[71].

An autosomal recessive modifier gene *mf*, which restricts the effect of *F*, has also been found in some chicken breeds [72], [73]. Pathogen free stocks are required for RCAS mediated gene misexpression. The available SPAFAS chickens are of White Leghorn. Landauer [72] indicated White Leghorns chickens are likely enriched for the recessive modifier of the frizzle phenotype. This may help explain the less impressive phenotypes we observed with misexpression of a virally-derived frizzle protein, although whether this is really the case remains an open question.

### Mammalian *KRT75* related mutations

In mammals, the α-keratin K75 (Keratin 75 or cytokeratin 75, formerly known as K6hf or hfK6) is a hair follicle-specific epithelial keratin [58]. K75 plays an essential role in hair and nail formation. The *KRT75* gene is specifically expressed in the companion layer [the cellular layer that lies between the outer (ORS) and inner (IRS) root sheaths], the upper germinative matrix region of the hair follicle (where mitosis takes place and hair keratins are produced), and the medulla of the hair shaft [74]–[78].

Mutations in *KRT75* have been associated with the hair disorderpseudofolliculitis barbae (PFB). Pseudofolliculitis barbae is a common human hair disorder characterized by a pustular foreign body inflammatory reaction that is induced by ingrown hairs of the facial and submental (barbea) regions after regular shaving [79], [80]. This abnormal hair orientation phenotype is somewhat similar to the frizzle feather we report here. Interestingly, an unusual Ala12Thr polymorphism in the 1A alpha-helical segment of K75 has been associated with PFB by examination of a three-generation Caucasian family as well as 100 individuals affected with PFB and 100 unaffected controls. Modeling and transfection studies led the investigators to conclude that the Ala12Thr substitution is disruptive at later stages of filament assembly and could represent one of the genetic factors leading to this complex phenotype. This abnormal hair orientation phenotype is somewhat similar to the frizzle feather we report here. Besides, another hair-follicle-specific epithelial keratin is also known to be associated with the autosomal dominant wooly hair syndrome [81], [82].

### Genomic aspects

A total of 54 functional keratin genes in the human genome can be divided into 28 type I genes and 26 type II genes [83], [84]. *KRT75* is located within the type II keratin gene cluster on chromosome 12 of humans and chromosome 15 of mice [85]. Twenty out of the 26 type II keratin genes are epithelial keratins and six encode hair keratins [80]. *KRT75* is tightly linked with epithelial keratin genes *KRT6A/B/C* and hair keratin genes *KRT81–86* in humans [83]. Mutations in human hair-follicle specific epithelial type II keratins are known to cause structural defects of differing severity in hair, nail, and skin [86], but the regulation of these keratins during proliferation and differentiation is yet to be elucidated. The chicken and zebra finch genomes contain only 28 and 27 α-keratin genes respectively compared to 41 genes in the anole genome [87]. β-keratin duplications occurred more frequently in birds than in reptiles [87] and they may have replaced some important roles in the formation of hard appendages in birds, thus the remaining α-keratins in the birds' genomes should play irreplaceable roles in the formation of epithelial and epidermal appendages of birds. The frizzle feather might represent a phenotype that could also be caused by mutations in genes involved in rachis structure but other than *KRT75*. Thus, this group of appendage structural mutants can be considered as a sub-category of ectodermal dysplasia. Interestingly, we observed that the rachis and barbs of feathers from homozygous frizzle chickens were easily broken during handling, and they were also easier to pluck. However, no abnormalities in nails were noted (unpublished observation). Further studies will provide clues into the architectural principles controlling how various skin appendages are built. It also calls for more molecular investigation into the role of this gene cluster in the evolution and development of the feather.

Another interesting phenomenon we report is the use of the cryptic splice site in this mutant. A cryptic splice site is a suppressed splice site that is recognized but usually not utilized by the splicing machinery until a mutation activates it, either by strengthening the cryptic splice site or disrupting an authentic splice site [88], [89]. Disruption of authentic splice sites crucial for identification of the 5′ or 3′ splice sites frequently result in complete exon skipping or in activating of the use of cryptic splice sites [90]. For genes with many introns, it is thought that up to 50% of mutations that cause disease actually affect splicing, either through the activation of cryptic splice sites, exon skipping, or disruption of alternative splicing [91]–[94].

In conclusion, we show that a single *KRT75* allele is the major determinant of frizzle feathers in chickens. It is most interesting to compare the phenotypes caused by a mutation in *KRT75*: pseudofollolliculitis barbae in human and frizzle feather in chicken. The phenotypes appear to be very different, but indeed fundamentally similar in that both exhibit appendage architecture defects. In chicken, the defect appears to be exaggerated due to the elaborate morphogenesis of feathers. Thus the ability to identify a gene that contributes to feather morphology highlights the potential contribution of chicken genetics to the understanding of feather variations. It also illustrates how the progress in chicken genomics provides a new approach to dissect basic biological questions [28], in this case, the molecular determinants of feather forms. Finally, this body of work highlights the importance of the cluster of α-keratin genes and invites further exploration into their role in normal and aberrant vertebrate developmental processes and provides an impetus for analyzing the relationship between α- and β-keratin during feather evolution.

## Materials and Methods

### Ethics statement

Animal care and experiments were conducted according to the guidelines established by the USC Institutional Animal Care and Use Committee.

### Animals and eggs

For the linkage and association mapping, DNA from blood or feathers was opportunistically obtained as a byproduct of National Poultry Improvement Plan testing. The first pedigree was derived from a bantam White-tailed Japanese/Silver Penciled Plymouth Rock frizzle heterozygote rooster and the second from a bantam Red Cochin frizzle heterozygote rooster. These males were crossed with 9 normal feathered bantam hens of the following breeds: Araucana, Barred Plymouth Rock, and White Leghorn. Eight to eighteen offspring were evaluated from each female. The first cross produced 33 frizzles and 29 wild-type and the second 19 frizzles and 27 wild-type for a total of 108 chicks. Frizzle embryos used in embryonic studies were generated by mating a homozygous frizzle rooster with homozygous frizzle hems. Embryos were collected at different embryonic stages. Feathers for image analysis were from sex-matched white Plymouth Rock siblings that were both heterozygous and homozygous for the *F* gene. Chicks were hatched and raised in the USC animal facility. For the functional study, pathogen free fertilized eggs were purchased from SPAFAS, Preston, CT. Some of these eggs hatched and the chickens were used for functional studies on adult feather follicles. Additional frizzle chickens were opportunistically sampled from farms in Wanhua and Tamsui, Taiwan.

### Image analyses of chicken feathers

Feathers were approximated as two-dimensional objects, which defined a primary plane for our imaging. Typical images of the feathers were taken from the dorsal side. We identified the fringes of the rachis by edge-finding algorithms we previously developed and defined the backbone of the rachis as a curve equidistant to the two fringes. The curve was smoothed by a length scale of 3 *mm* to suppress noises due to image errors. We parameterized this backbone by *s*, the accumulated distance from the proximal end that is defined as the origin (*s* = 0). The total length of the rachis means the accumulated distance from the origin to the distal end. We described the bending of the rachis by a function **θ**(*s*), in which **θ** is an angle (with an arbitrary reference) representing the tangent of the backbone at the location *s*.

### Paraffin section and staining

The generated or gene misexpressed feathers were fixed in 4% paraformaldehyde at 4°C overnight followed by procedures described by Jiang et al. (1998) for immunohistochemistry and 7 µm paraffin sections were prepared [95]. PCNA and AMV-3C2 antibodies are from Chemicon (CBL407) and Hybridoma Bank respectively. Double fluorescent immunostaining was done using K75 antibody (ab76486; Abcam, MA) and feather keratin antibody from Dr. Roger Sawyer. Section were imaged with a Zeiss 510 confocal microscope (University of Southern California Liver Center). DAPI was used to visualize the nuclei. TUNEL assay was preformed according to the protocol provided by Millipore (catalog number S7101).

### mRNA *in situ* hybridization

We performed PCR for the full length chicken *KRT75* by using sense primer (5′-ATGTCTCGCCAGTCCACCG-3′) and antisense primer (5′-TTAGCTCCTGTAACTTCTCC-3′. The PCR product was inserted into the p-drive plasmid (Qiagen). Antisense probe was made to detect the *KRT75* mRNA by section or whole mount *in situ* hybridization. Non-radioactive *in situ* hybridization was performed according to procedures described by Chuong et al (1996) [96]. *SHH* antisense probe was generated as previously described [49]. Feather keratin antisense probe was used to detect feather keratin B [97].

### Linkage analysis

In order to locate the gene underlying the frizzle trait, a genome scan was conducted on progeny of crosses between the same heterozygous frizzle rooster, PF1, and five different wild-type feathered hens. A total of 2678 SNPs were genotyped using the Illumina Goldengate assay. The average genotype call rate obtained for the 45 birds in the study was 99.37% (range 98.32–99.74%) providing approximately 2661 genotypes per bird. The genotype data was screened for Mendelian incompatibilities using PEDCHECK, while MERLIN was used to assess the data for occurrence of double recombination events over short genetic distances, which are most likely due to genotyping error. MLINK of the FASTLINK package was used to perform two-point linkage analysis. An autosomal dominant mode of inheritance with complete penetrance and a mutant allele frequency of 0.001 was used in the analysis.

### Positional cloning and genotyping

Chicken genomic DNA was isolated from blood using the Blood & Cell Culture DNA Mini Kit (Qiagen, Hilden, Germany). For mutation analysis of the keratin candidate genes, we got 49 PCR amplicons of the selected candidates amplified from chicken genomic DNA (Table S1). Primers to cover some intronic and exonic regions of 14 keratin genes were designed using the CLC Bio 6.0 (Aarhus, Denmark). All amplicons were sequenced directly after treatment with exonuclease I and shrimp alkaline phosphatase by standard methods. Each amplicon was sequenced using BigDye terminator sequencing kits and standard protocols (Applied Biosystems, Santa Clara, CA). A significant mutation was detected in only one amplicon amplified by a pair of primers (5′-CCATGGACAACAACCGCAAC-3′ and 5′-TTTCCTTCCTTCCTTCCAATCCT-3′).

### RT–PCR

Feather follicle samples were collected from a homozygous frizzle chicken 2 weeks after plucking and then were immersed in RNALater (Ambion, Austin, TX) and stored at −20°C. After thawing, total RNA was isolated by homogenization and extraction using the RNeasy Tissue Midi kit (Qiagen, Hilden, Germany). Each RT-PCR reaction was carried out with 1 ug of total RNA. Primers were designed for the *KRT75* gene (5′-TTTCTTCTTTCCCTCCCACT-3′ and 5′- GTTCTGCTTCCCCTGATTAT-3′).

### Generation of proviral constructs

*KRT75* cDNA PCR products were cloned into the pCR8/GW/TOPO Gateway entry vector (Invitrogen, Carlsbad, CA) and sequenced. An LR recombination reaction was performed to transfer the cDNAs to a Gateway compatible RCASBP-Y DV vector [98].

### Construction of RCAS-KRT75-WT and RCAS-KRT75-MT

*KRT75* wildtype (KRT75-WT) and *KRT75* mutant forms (KRT75-MT) were cloned to RCAS by the Gateway system (Invitrogen, Carlsbad, CA). Virus was made according to Jiang et al., 1998 [95] and concentrated by ultra-centrifugation.

### Feather regeneration

Around 30 contour feathers from the middle back of the body were plucked and then 10 days or 30 days were allowed to pass. At the collection points, regenerated feathers were directly plucked or dissected and the whole single follicles were prepared for sectioning.

### Functional studies of *KRT75*

For embryonic studies, RCAS-KRT75-WT or RCAS-KRT75-MT virus was injected into the amniotic cavity of E3 chicken embryos. Samples were collected at E13. RCAS-GFP was injected into different embryos as a control. For adult feathers, about 100 µl of virus was injected into the empty follicles after plucking the primary flight feathers in the left wing. The feathers on the right wing were collected at same time as controls. Feather follicles from a different chicken injected with RCAS-GFP were used as an alternative control. Feather morphogenesis was observed after 1–2 months of regeneration.

### Expression of *KRT75* in mammalian cells

PtK2 cells (American Tissue Culture Collection, MD) were transfected with plasmid (RCAS) encoding KRT75-WT or KRT75-MT by lipofection (Lipofectamine, Invitrogen, CA). After 48 hours samples were fixed in 4% paraformaldehyde and stained with antibodies to K18 (AV40206; Sigma, MO) and K75. DAPI was used to visualize the nuclei. Images were obtained using a Zeiss 510 confocal microscope.

### Accession number

The sequence of *F* allele of *KRT75* has been submitted to GenBank with the accession number JQ013796.

## Supporting Information

Figure S1 Detailed PCNA and TUNEL staining in the rachis of normal and frizzle chicken regenerated body feathers at different levels. (A) Diagram of a 30 day regenerating follicle. Levels I, II and III show the planes of section, from immature to mature regions of a feather follicle. (B, C) Comparison of PCNA staining between normal (B) and frizzle (C) feathers at different levels. (D, E). Comparison of TUNEL staining between normal (D) and frizzle (E) feathers at different levels. (F) Diagram to summarize the PCNA (red) and TUNEL (green) data. D, dorsal; DP, dermal papilla; V, ventral.
(TIF)

Figure S2 Schematic of the *F* critical region. (A) The region within the chicken linkage group E22C19W28_E50C23 harbouring the *F* mutation in the *KRT75* gene. (adapted from the Ensembl genome browser http://www.ensembl.org/). (B) The *F* mutation was determined to be an 84-bp deletion covering the junction of exon 5 and intron 5 in the *KRT75* gene and is indicated by a yellow bar. The deletion activates a cryptic splice site in exon 5.
(TIF)

Figure S3 *KRT75* genotypes of parental chickens and progenies in the experimental cross. These results were obtained with DNA samples used for the genome-scan and those that were not included in the genome scan P. (A) Lab IDs, phenotypes and lane number for samples used in the genome-scan. (B) *KRT75* genotypes of samples used in the genome scan. Samples 33 and 39–41 are represented by longer exposure times. (C) Lab IDs, phenotypes and lane numbers for samples not used in the genome scan. (D) KRT75 genotypes of samples not used in the genome scan. Samples 78, 81, 89 and 96 represent repeat PCR reactions. A total of 96 PCR reactions were performed and include 2 heterozygous frizzle phenotype roosters, 9 homozygous wild type hens, 38 frizzle phenotype progeny and 47 wild type phenotype progeny. In each case, the genotype showed perfect correlation with the phenotype. F, frizzle phenotype; M, DNA marker; W, wild type.
(TIF)

Figure S4 The summary of *KRT75* genotypes of the parents and progenies in the experimental cross. Lane 1 shows the genotype of the heterozygous father (+/*F*), lane 2 shows the genotype of wild-type mother, lane 3 shows the genotype of frizzle offsprings, lane 4 show the genotype of normal offspring (I do not think we need delete this)
(TIF)

Figure S5 *KRT75* is expressed in the frizzle feather follicles. Lanes 1–3 show the [72], [73]PCR products amplified from total cDNA prepared using a poly-T oligonucleotide primer, whereas lanes 4–6 show the PCR products amplified from total cDNA prepared using random hexamer primers. Lanes 1 and 4 show the PCR products amplified using primers located in the 5′- and 3′-UTRs of *KRT75* mRNA (5′-TTTCTTCTTTCCCTCCCACT-3′ and 5′- GTTCTGCTTCCCCTGATTAT-3′), whereas lanes 2, 3, 5, and 6 show the PCR products containing the complete CDS only (5′-ATGTCTCGCCAGTCCACCG-3′ and 5′-TTAGCTCCTGTAACTTCTCC-3′).
(TIF)

Figure S6 Multiple sequence alignment of amniote K75 proteins. The alignment was prepared using MUSCLE implemented in the CLC Bio 6.0 (Aarhus, Denmark). The sequences were translated from AY574985.1 (red junglefowl), XM_003205996 (turkey), XM_002194971 (zebrafinch KRT6A), XM_002194995 (zebrafinch *KRT75*), XM_003216971 (lizard), XM_001362788 (opossum), XM_001504398 (horse), BC137935 (mouse), and NM_004693 (human). The sequence of *F* allele of *KRT75* has been submitted to GenBank with the accession number JQ013796.
(TIF)

Figure S7 Domains of K75. The yellow-line box highlights the deletion which removes the entire part of link L2 and some parts of the coiled-coil segments of 2A and 2B. The domain information was obtained from the Human Intermediate Filament Mutation Database (www.interfil.org).
(TIF)

Figure S8 Detailed H&E and TUNEL staining at different levels among control, RCAS-KRT75-WT and RCAS-KRT75-MT mis-expression samples. (A) Diagram showing levels for section from immature (level I) to mature (level IV) regions of an E13 embryonic body feather. (B–D′) H&E and TUNEL staining at different levels. (B) and (B′) control; (C) and (C′) RCAS-KRT75-WT; (D) and (D′) RCAS-KRT75-MT. PtK2 cells stained for KRT18 (red), K75 (green) and DAPI (blue). (E) Wildtype cells; (F) RCAS-KRT75-WT; (G) RCAS-KRT75-MT.
(TIF)

Figure S9 Natural bending of feathers from both sides of a chicken. (A) Images of flight feathers from opposite sides of the same normal chicken (the R8 and L8 denote the 8th feather taken from the right and left wing, respectively). (B) The trend of curves (*s*) shows reflective symmetry.
(TIF)

Figure S10 Contrast between feathers that have misexpressed GFP and overexpressed KRT75-WT. (A) Comparison of the feathers with misexpressed GFP (Control) and KRT75-WT. (B) Effects of the viral misexpression, as shown by the qualitative change of the curves of θ(*s*).
(TIF)

Table S1 Primers designed to screen for the causative mutation in the candidate gene regions.
(PDF)

Table S2 Sequence variants within the linkage group chrE22C19W28_E50C23.
(PDF)

## References

[1] Darwin C (1868). The Variation of Plants and Animals under Domestication.

[2] Bartels T (2003). Variations in the morphology, distribution, and arrangement of feathers in domesticated birds.. J Exp Zool B Mol Dev Evol.

[3] Widelitz RB, Veltmaat JM, Mayer JA, Foley J, Chuong CM (2007). Mammary glands and feathers: comparing two skin appendages which help define novel classes during vertebrate evolution.. Semin Cell Dev Biol.

[4] Lin CM, Jiang TX, Widelitz RB, Chuong CM (2006). Molecular signaling in feather morphogenesis.. Curr Opin Cell Biol.

[5] Wu P, Hou L, Plikus M, Hughes M, Scehnet J (2004). Evo-Devo of amniote integuments and appendages.. Int J Dev Biol.

[6] Yu M, Yue Z, Wu P, Wu DY, Mayer JA (2004). The developmental biology of feather follicles.. Int J Dev Biol.

[7] Widelitz RB, Jiang TX, Yu M, Shen T, Shen JY (2003). Molecular biology of feather morphogenesis: a testable model for evo-devo research.. J Exp Zool B Mol Dev Evol.

[8] Chuong CM, Homberger DG (2003). Development and evolution of the amniote integument: current landscape and future horizon.. J Exp Zool B Mol Dev Evol.

[9] Yu M, Wu P, Widelitz RB, Chuong CM (2002). The morphogenesis of feathers.. Nature.

[10] Chuong CM, Chodankar R, Widelitz RB, Jiang TX (2000). Evo-devo of feathers and scales: building complex epithelial appendages.. Curr Opin Genet Dev.

[11] Prum RO (2005). Evolution of the morphological innovations of feathers.. J Exp Zool B Mol Dev Evol.

[12] Prum RO, Dyck J (2003). A hierarchical model of plumage: morphology, development, and evolution.. J Exp Zool B Mol Dev Evol.

[13] Prum RO, Brush AH (2002). The evolutionary origin and diversification of feathers.. Q Rev Biol.

[14] Prum RO, Williamson S (2001). Theory of the growth and evolution of feather shape.. J Exp Zool.

[15] Prum RO (1999). Development and evolutionary origin of feathers.. J Exp Zool.

[16] Sawyer RH, Rogers L, Washington L, Glenn TC, Knapp LW (2005). Evolutionary origin of the feather epidermis.. Dev Dyn.

[17] Sawyer RH, Knapp LW (2003). Avian skin development and the evolutionary origin of feathers.. J Exp Zool B Mol Dev Evol.

[18] Somes RG, Crawford RD (1990). Mutations and major variants of plumage and skin in chickens.. Poultry Breeding and Genetics.

[19] Lucas AM, Stettenheim PR (1972). Avian Anatomy - Integument, Parts I and II.

[20] Aerts J, Crooijmans R, Cornelissen S, Hemmatian K, Veenendaal T (2003). Integration of chicken genomic resources to enable whole-genome sequencing.. Cytogenet Genome Res.

[21] Antin PB (2004). Chicken genomic and gene expression resources.. FASEB J.

[22] Antin PB, Konieczka JH (2005). Genomic resources for chicken.. Dev Dyn.

[23] Burt D, Pourquie O (2003). Genetics. Chicken genome–science nuggets to come soon.. Science.

[24] Burt DW (2002). Comparative mapping in farm animals.. Brief Funct Genomic Proteomic.

[25] Burt DW (2004). Chicken genomics charts a path to the genome sequence.. Brief Funct Genomic Proteomic.

[26] Burt DW (2004). The chicken genome and the developmental biologist.. Mech Dev.

[27] Burt DW (2007). Emergence of the chicken as a model organism: Implications for agriculture and biology.. Poult Sci.

[28] Burt DW, White SJ (2007). Avian genomics in the 21st century.. Cytogenet Genome Res.

[29] Cogburn LA, Porter TE, Duclos MJ, Simon J, Burgess SC (2007). Functional genomics of the chicken - A model organism.. Poult Sci.

[30] de Koning DJ, Cabrera CP, Haley CS (2007). Genetical genomics: Combining gene expression with marker genotypes in poultry.. Poultry Sci.

[31] Delany ME (2004). Genetic variants for chick biology research: from breeds to mutants.. Mech Dev.

[32] Dequeant ML, Pourquie O (2005). Chicken genome: new tools and concepts.. Dev Dyn.

[33] Dodgson JB (2003). Chicken genome sequence: a centennial gift to poultry genetics.. Cytogenet Genome Res.

[34] Edwards SV, Bryan Jennings W, Shedlock AM (2005). Phylogenetics of modern birds in the era of genomics.. Proc Biol Sci.

[35] Burt DW (2005). Chicken genome: current status and future opportunities.. Genome Res.

[36] Ellegren H (2005). The avian genome uncovered.. Trends Ecol Evol.

[37] Ellegren H (2007). Molecular evolutionary genomics of birds.. Cytogenet Genome Res.

[38] Anderson KV, Ingham PW (2003). The transformation of the model organism: a decade of developmental genetics.. Nat Genet.

[39] Andersson L (2001). Genetic dissection of phenotypic diversity in farm animals.. Nat Rev Genet.

[40] Andersson L, Georges M (2004). Domestic-animal genomics: deciphering the genetics of complex traits.. Nat Rev Genet.

[41] Wright D, Boije H, Meadows JR, Bed'hom B, Gourichon D (2009). Copy number variation in intron 1 of SOX5 causes the Pea-comb phenotype in chickens.. PLoS Genet.

[42] Wright D, Kerje S, Brandstrom H, Schutz K, Kindmark A (2008). The genetic architecture of a female sexual ornament.. Evolution.

[43] Somes RG, Crawford RD (1990). Mutations and major variants of muscles and skeleton in Guinea Fowl.. Poultry Breeding and Genetics.

[44] Landauer W, Dunn LC (1930). The “Frizzle” character of fowls - Its expression and inheritance.. J Hered.

[45] Hutt FB (1930). The genetics of the fowl I The inheritance of frizzled plumage.. J Genet.

[46] Winter H, Rogers MA, Langbein L, Stevens HP, Leigh IM (1997). Mutations in the hair cortex keratin hHb6 cause the inherited hair disease monilethrix.. Nat Genet.

[47] Chapalain V, Winter H, Langbein L, Le Roy JM, Labreze C (2002). Is the loose anagen hair syndrome a keratin disorder? A clinical and molecular study.. Arch Dermatol.

[48] Chen J, Jaeger K, Den Z, Koch PJ, Sundberg JP (2008). Mice expressing a mutant Krt75 (K6hf) allele develop hair and nail defects resembling pachyonychia congenita.. J Invest Dermatol.

[49] Ting-Berreth SA, Chuong CM (1996). Sonic Hedgehog in feather morphogenesis: induction of mesenchymal condensation and association with cell death.. Dev Dyn.

[50] Maderson PF, Hillenius WJ, Hiller U, Dove CC (2009). Towards a comprehensive model of feather regeneration.. J Morphol.

[51] Alibardi L, Toni M (2008). Cytochemical and molecular characteristics of the process of cornification during feather morphogenesis.. Prog Histochem Cytochem.

[52] Krimm S (1960). Structure of Frizzle Mutant Feather Keratin.. J Mol Biol.

[53] Brush AH (1972). Correlation of protein electrophoretic pattern with morphology of normal and mutant feathers.. Biochem Genet.

[54] Szeverenyi I, Cassidy AJ, Chung CW, Lee BT, Common JE (2008). The Human Intermediate Filament Database: comprehensive information on a gene family involved in many human diseases.. Hum Mutat.

[55] Parry DA, Steinert PM (1999). Intermediate filaments: molecular architecture, assembly, dynamics and polymorphism.. Q Rev Biophys.

[56] Wang Z, Wong P, Langbein L, Schweizer J, Coulombe PA (2003). Type II epithelial keratin 6hf (K6hf) is expressed in the companion layer, matrix, and medulla in anagen-stage hair follicles.. J Invest Dermatol.

[57] Mou C, Pitel F, Gourichon D, Vignoles F, Tzika A (2011). Cryptic patterning of avian skin confers a developmental facility for loss of neck feathering.. PLoS Biol.

[58] Schweizer J, Bowden PE, Coulombe PA, Langbein L, Lane EB (2006). New consensus nomenclature for mammalian keratins.. J Cell Biol.

[59] Moll R, Divo M, Langbein L (2008). The human keratins: biology and pathology.. Histochem Cell Biol.

[60] McLean WH, Moore CB (2011). Keratin disorders: from gene to therapy.. Hum Mol Genet.

[61] Fuchs E, Tyner AL, Giudice GJ, Marchuk D, Raychaudhury A (1987). The Human Keratin Genes and Their Differential Expression.. Curr Top Dev Biol.

[62] Fuchs E, Cleveland DW (1998). A structural scaffolding of intermediate filaments in health and disease.. Science.

[63] Dhouailly D, Rogers GE, Sengel P (1978). The specification of feather and scale protein synthesis in epidermal-dermal recombinations.. Dev Biol.

[64] Haake AR, Konig G, Sawyer RH (1984). Avian feather development: relationships between morphogenesis and keratinization.. Dev Biol.

[65] Bell E, Thathachari YT (1963). Development of feather keratin during embryogenesis of the chick.. J Cell Biol.

[66] Chang CH, Yu M, Wu P, Jiang TX, Yu HS (2004). Sculpting skin appendages out of epidermal layers via temporally and spatially regulated apoptotic events.. J Invest Dermatol.

[67] Tong X, Coulombe PA (2006). Keratin 17 modulates hair follicle cycling in a TNFalpha-dependent fashion.. Genes Dev.

[68] Landauer W, Aberle SD (1935). Studies on the endocrine glands of Frizzle fowl.. Am J Anat.

[69] Benedict FG, Landauer W, Fox EL (1932). The physiology of normal and frizzle fowl, with special reference to the basal metabolism.. Storrs Agric Exp Sta Bull.

[70] Boas EP, Landauer W (1933). The effect of elevated metabolism on the hearts of frizzle fowl.. Am J Med Sci.

[71] Landauer W, Upham E (1936). Weight and size in frizzle fowl.. Storrs Agric Exp Sta Bull.

[72] Landauer W (1933). A gene modifying frizzling in the fowl.. J Hered.

[73] Hutt FB (1936). Genetics of the fowl V The modified frizzle.. J Genet.

[74] Sperling LC, Hussey S, Sorrells T, Wang JA, Darling T (2010). Cytokeratin 75 expression in central, centrifugal, cicatricial alopecia - new observations in normal and diseased hair follicles.. J Cutan Pathol.

[75] Winter H, Langbein L, Praetzel S, Jacobs M, Rogers MA (1998). A novel human type II cytokeratin, K6hf, specifically expressed in the companion layer of the hair follicle.. J Invest Dermatol.

[76] Langbein L, Spring H, Rogers MA, Praetzel S, Schweizer J (2004). Hair keratins and hair follicle-specific epithelial keratins.. Methods Cell Biol.

[77] Coulombe PA, Wang ZL, Wong P, Langbein L, Schweizer J (2003). Type II epithelial keratin 6hf (K6hf) is expressed in the companion layer, matrix, and medulla in anagen-stage hair follicles.. J Invest Dermatol.

[78] Gu LH, Coulombe PA (2007). Keratin expression provides novel insight into the morphogenesis and function of the companion layer in hair follicles.. J Invest Dermatol.

[79] Winter H, Schissel D, Parry DA, Smith TA, Liovic M (2004). An unusual Ala12Thr polymorphism in the 1A alpha-helical segment of the companion layer-specific keratin K6hf: evidence for a risk factor in the etiology of the common hair disorder pseudofolliculitis barbae.. J Invest Dermatol.

[80] Schweizer J, Langbein L, Rogers MA, Winter H (2007). Hair follicle-specific keratins and their diseases.. Exp Cell Res.

[81] Shimomura Y, Wajid M, Petukhova L, Kurban M, Christiano AM (2010). Autosomal-dominant woolly hair resulting from disruption of keratin 74 (KRT74), a potential determinant of human hair texture.. Am J Hum Genet.

[82] Wasif N, Naqvi SK, Basit S, Ali N, Ansar M (2011). Novel mutations in the keratin-74 (KRT74) gene underlie autosomal dominant woolly hair/hypotrichosis in Pakistani families.. Hum Genet.

[83] Rogers MA, Edler L, Winter H, Langbein L, Beckmann I (2005). Characterization of new members of the human type II keratin gene family and a general evaluation of the keratin gene domain on chromosome 12q13.13.. J Invest Dermatol.

[84] Rogers MA, Winter H, Langbein L, Bleiler R, Schweizer J (2004). The human type I keratin gene family: characterization of new hair follicle specific members and evaluation of the chromosome 17q21.2 gene domain.. Differentiation.

[85] Hesse M, Zimek A, Weber K, Magin TM (2004). Comprehensive analysis of keratin gene clusters in humans and rodents.. Eur J Cell Biol.

[86] Smith F (2003). The molecular genetics of keratin disorders.. Am J Clin Dermatol.

[87] Vandebergh W, Bossuyt F (2012). Radiation and Functional Diversification of Alpha Keratins during Early Vertebrate Evolution.. Mol Biol Evol.

[88] Padgett RA, Grabowski PJ, Konarska MM, Seiler S, Sharp PA (1986). Splicing of messenger RNA precursors.. Annu Rev Biochem.

[89] Green MR (1986). Pre-mRNA splicing.. Annu Rev Genet.

[90] Krainer AR, Roca X, Sachidanandam R (2003). Intrinsic differences between authentic and cryptic 5 ′ splice sites.. Nucleic Acids Res.

[91] Krainer AR, Hastings ML, Resta N, Traum D, Stella A (2005). An LKB1 AT-AC intron mutation causes Peutz-Jeghers syndrome via splicing at noncanonical cryptic splice sites.. Nat Struct Mol Biol.

[92] Lopez-Bigas N, Audit B, Ouzounis C, Parra G, Guigo R (2005). Are splicing mutations the most frequent cause of hereditary disease?. FEBS Lett.

[93] Buratti E, Chivers M, Kralovicova J, Romano M, Baralle M (2007). Aberrant 5′ splice sites in human disease genes: mutation pattern, nucleotide structure and comparison of computational tools that predict their utilization.. Nucleic Acids Res.

[94] Wang GS, Cooper TA (2007). Splicing in disease: disruption of the splicing code and the decoding machinery.. Nat Rev Genet.

[95] Jiang T-X, Stott S, Widelitz RB, Chuong C-M, Chuong C-M (1998). Current methods in the study of avian skin appendages.. Molecular Basis of Epithelial Appendage Morphogenesis.

[96] Chuong CM, Widelitz RB, Ting-Berreth S, Jiang TX (1996). Early events during avian skin appendage regeneration: dependence on epithelial-mesenchymal interaction and order of molecular reappearance.. J Invest Dermatol.

[97] Presland RB, Whitbread LA, Rogers GE (1989). Avian keratin genes. II. Chromosomal arrangement and close linkage of three gene families.. J Mol Biol.

[98] Loftus SK, Larson DM, Watkins-Chow D, Church DM, Pavan WJ (2001). Generation of RCAS vectors useful for functional genomic analyses.. DNA Res.

