# Supplementary figures and images for: The Chicken Frizzle Feather Is Due to an α-Keratin (KRT75) Mutation That Causes a Defective Rachis
Source: PLoS Genet. 2012 Jul 19;8(7):e1002748. doi: 10.1371/journal.pgen.1002748 (PMC3400578; doi:10.1371/journal.pgen.1002748)

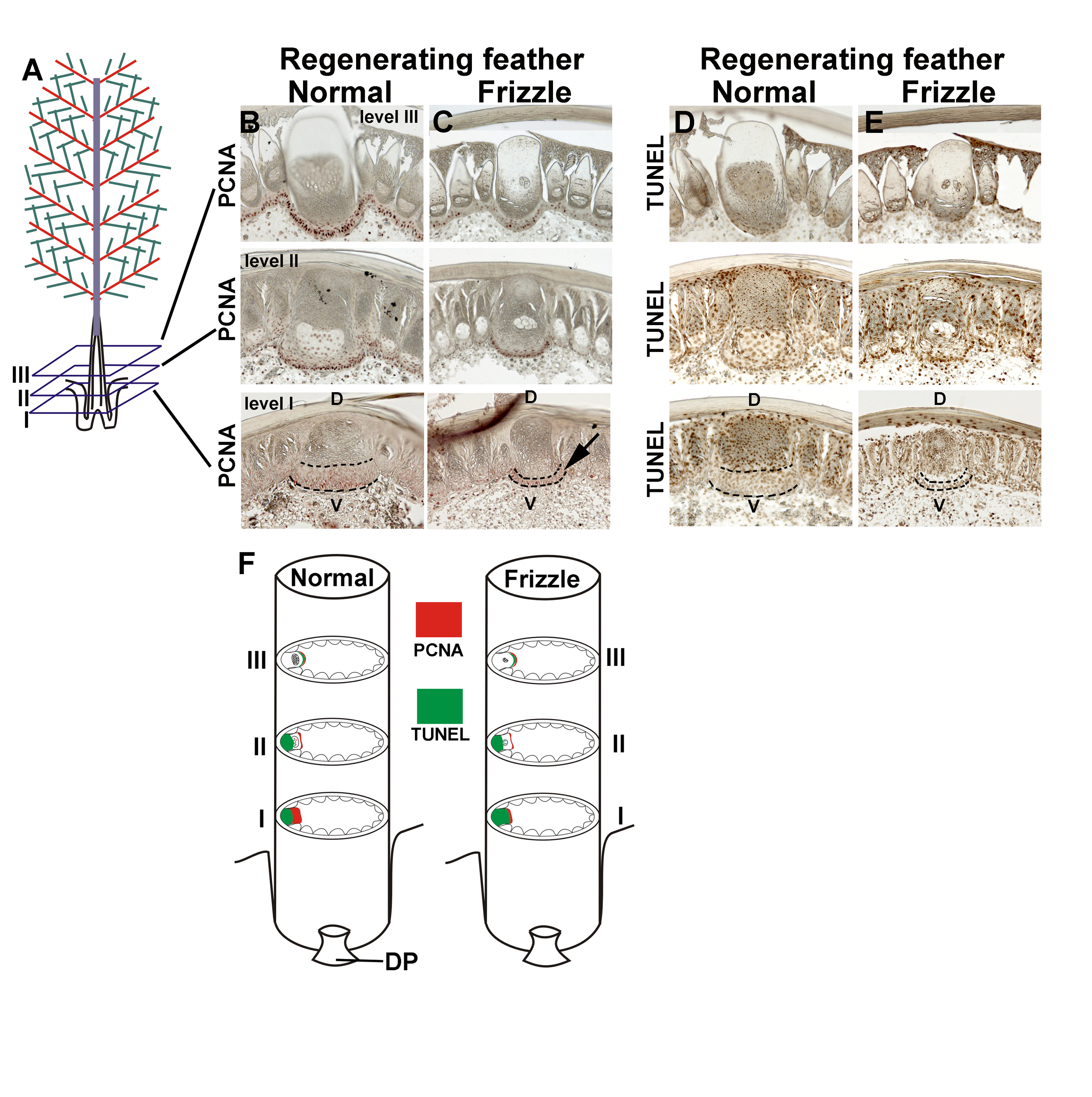

Supplement: Figure S1 — Detailed PCNA and TUNEL staining in the rachis of normal and frizzle chicken regenerated body feathers at different levels. (A) Diagram of a 30 day regenerating follicle. Levels I, II and III show the planes of section, from immature to mature regions of a feather follicle. (B, C) Comparison of PCNA staining between normal (B) and frizzle (C) feathers at different levels. (D, E). Comparison of TUNEL staining between normal (D) and frizzle (E) feathers at different levels. (F) Diagram to summarize the PCNA (red) and TUNEL (green) data. D, dorsal; DP, dermal papilla; V, ventral. (TIF) [file pgen.1002748.s001.tif]

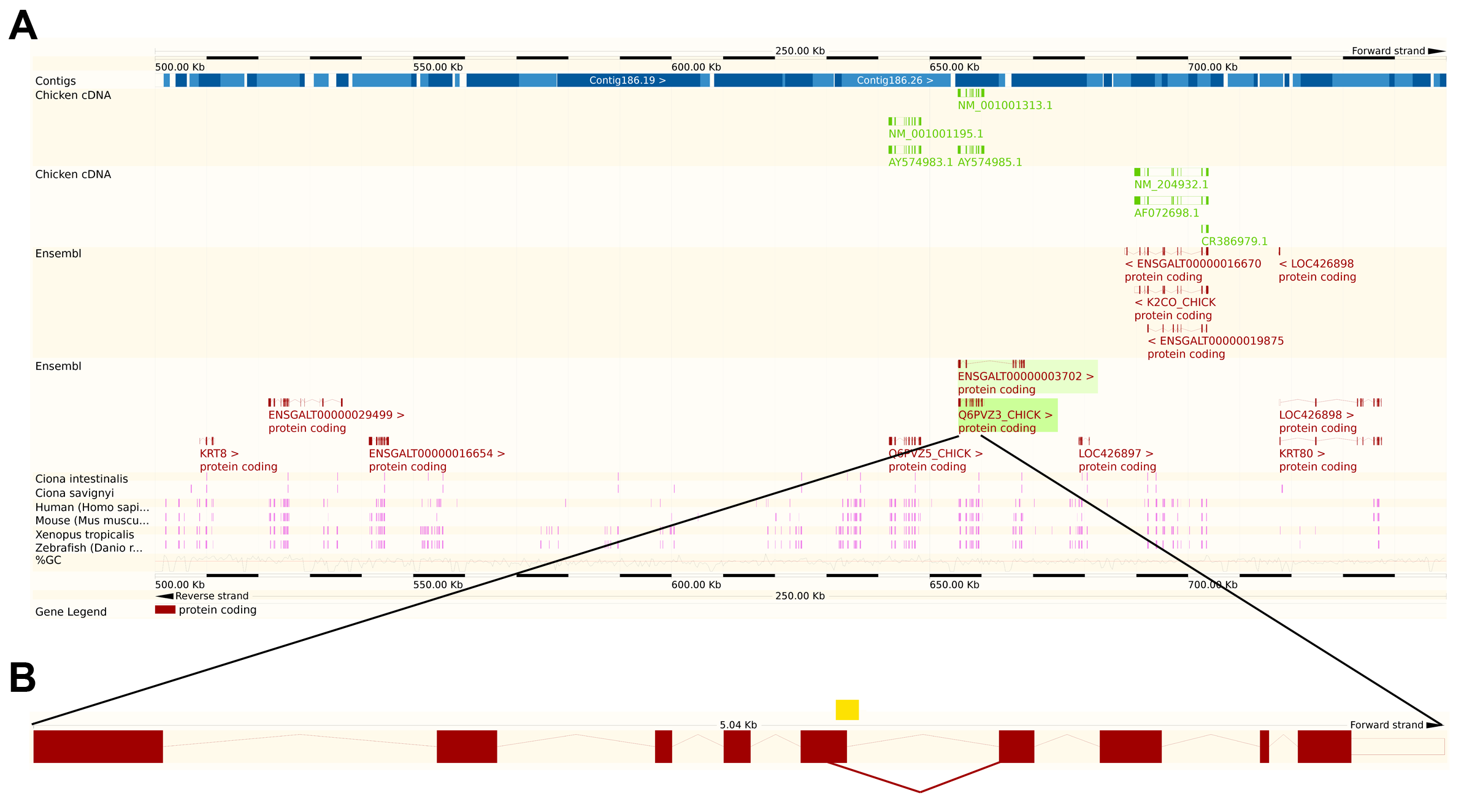

Supplement: Figure S2 — Schematic of the F critical region. (A) The region within the chicken linkage group E22C19W28_E50C23 harbouring the F mutation in the KRT75 gene. (adapted from the Ensembl genome browser http://www.ensembl.org/). (B) The F mutation was determined to be an 84-bp deletion covering the junction of exon 5 and intron 5 in the KRT75 gene and is indicated by a yellow bar. The deletion activates a cryptic splice site in exon 5. (TIF) [file pgen.1002748.s002.tif]

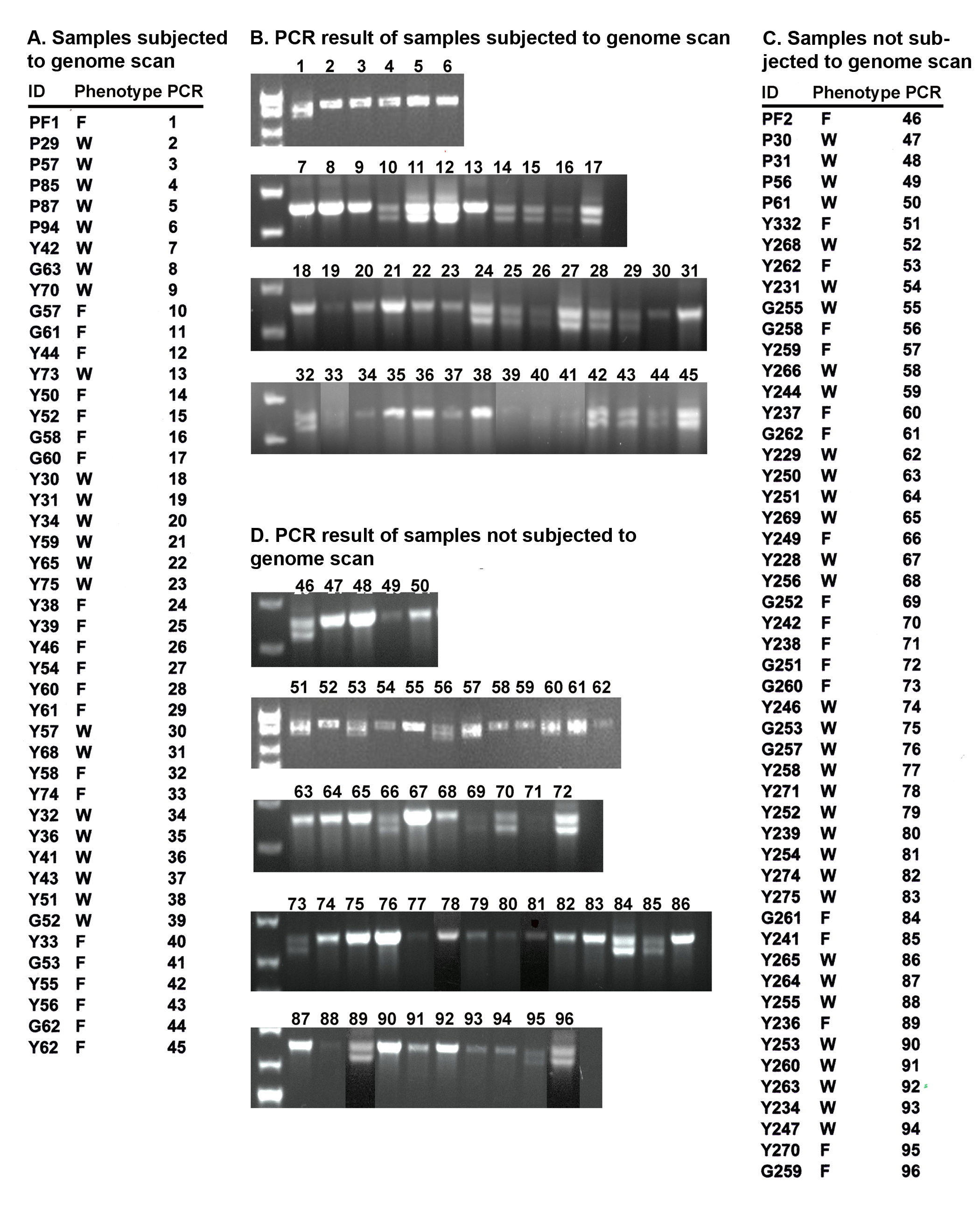

Supplement: Figure S3 — KRT75 genotypes of parental chickens and progenies in the experimental cross. These results were obtained with DNA samples used for the genome-scan and those that were not included in the genome scan P. (A) Lab IDs, phenotypes and lane number for samples used in the genome-scan. (B) KRT75 genotypes of samples used in the genome scan. Samples 33 and 39–41 are represented by longer exposure times. (C) Lab IDs, phenotypes and lane numbers for samples not used in the genome scan. (D) KRT75 genotypes of samples not used in the genome scan. Samples 78, 81, 89 and 96 represent repeat PCR reactions. A total of 96 PCR reactions were performed and include 2 heterozygous frizzle phenotype roosters, 9 homozygous wild type hens, 38 frizzle phenotype progeny and 47 wild type phenotype progeny. In each case, the genotype showed perfect correlation with the phenotype. F, frizzle phenotype; M, DNA marker; W, wild type. (TIF) [file pgen.1002748.s003.tif]

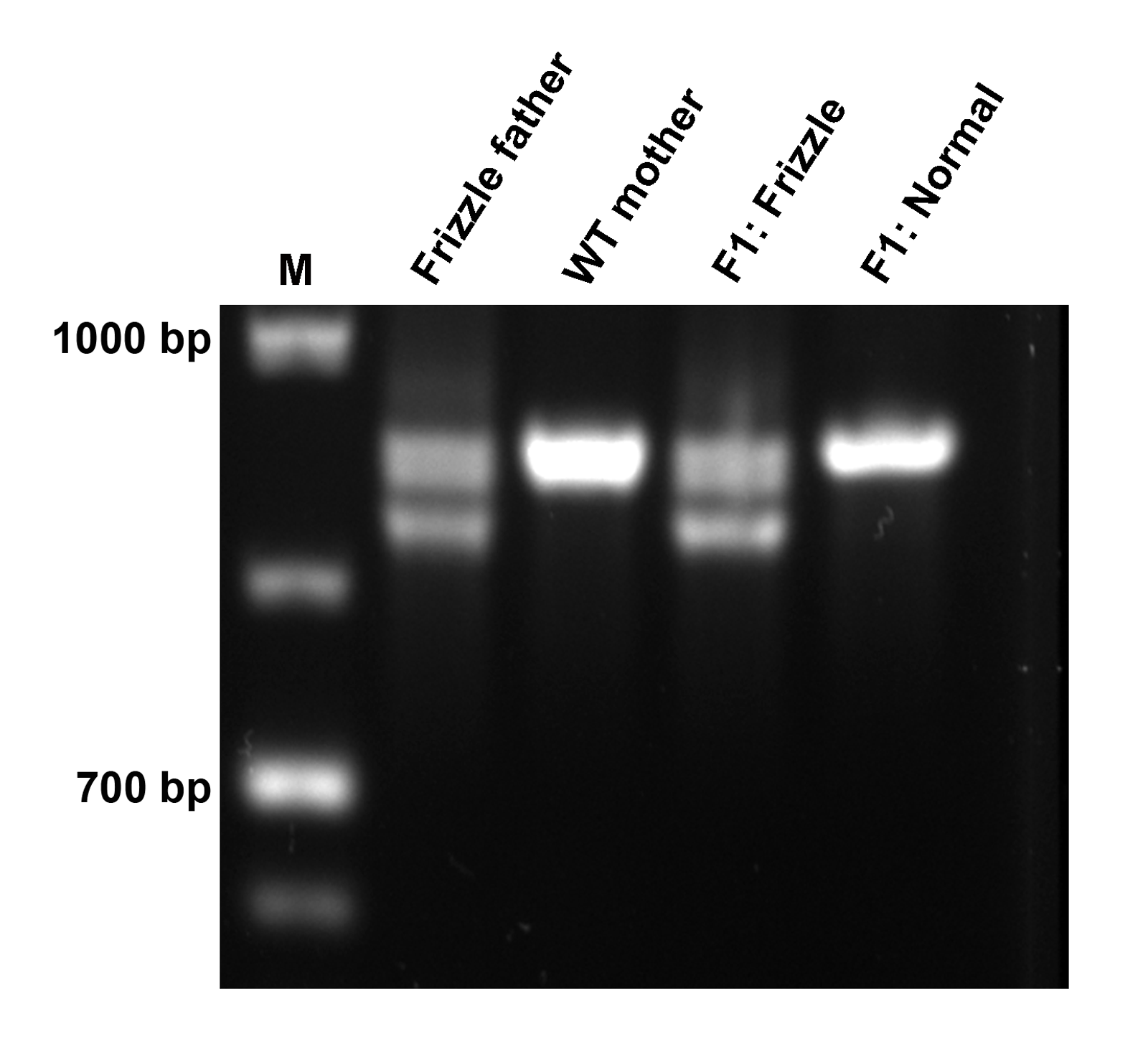

Supplement: Figure S4 — The summary of KRT75 genotypes of the parents and progenies in the experimental cross. Lane 1 shows the genotype of the heterozygous father (+/F), lane 2 shows the genotype of wild-type mother, lane 3 shows the genotype of frizzle offsprings, lane 4 show the genotype of normal offspring (I do not think we need delete this) (TIF) [file pgen.1002748.s004.tif]

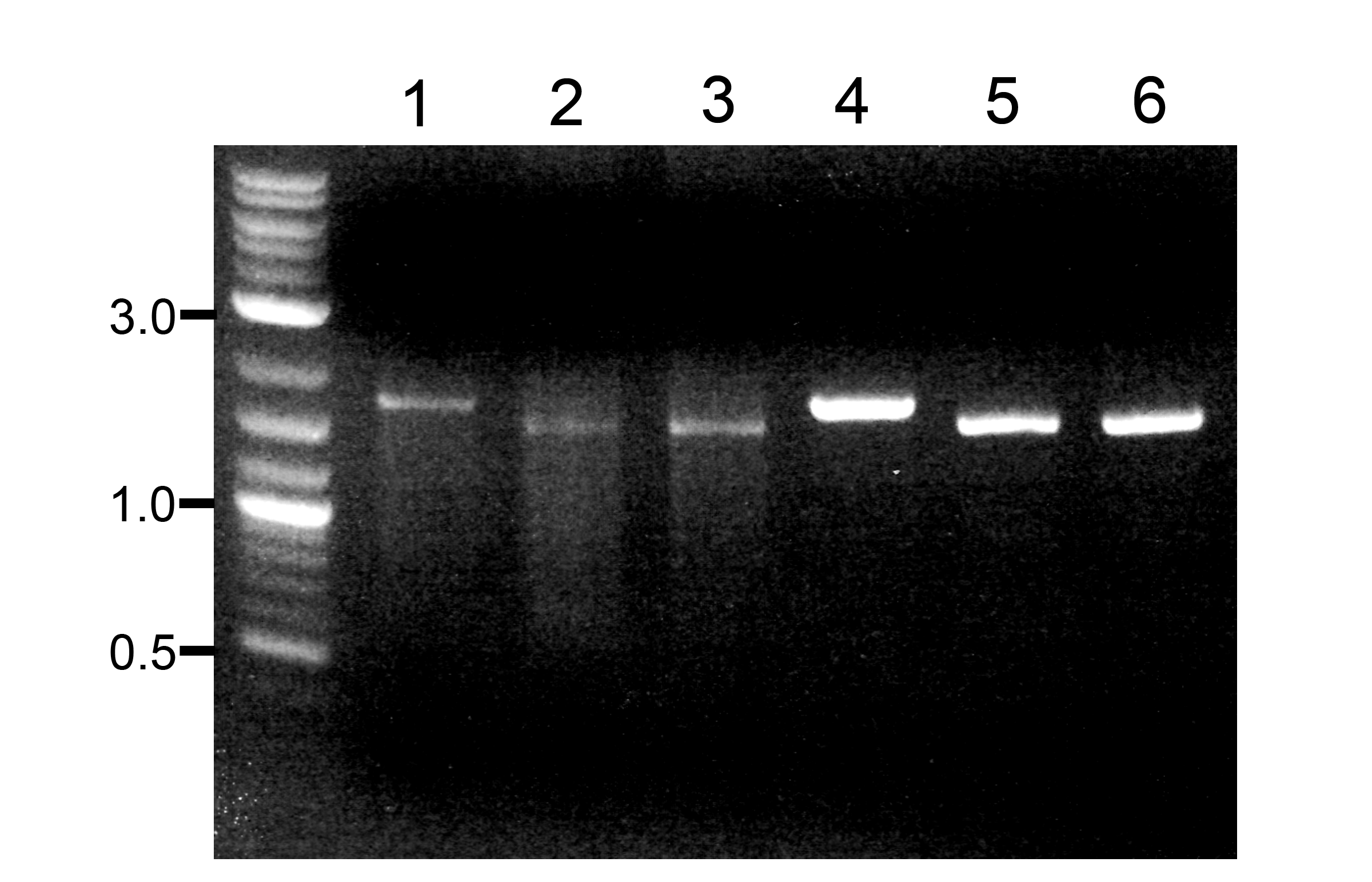

Supplement: Figure S5 — KRT75 is expressed in the frizzle feather follicles. Lanes 1–3 show the [72], [73]PCR products amplified from total cDNA prepared using a poly-T oligonucleotide primer, whereas lanes 4–6 show the PCR products amplified from total cDNA prepared using random hexamer primers. Lanes 1 and 4 show the PCR products amplified using primers located in the 5′- and 3′-UTRs of KRT75 mRNA (5′-TTTCTTCTTTCCCTCCCACT-3′ and 5′- GTTCTGCTTCCCCTGATTAT-3′), whereas lanes 2, 3, 5, and 6 show the PCR products containing the complete CDS only (5′-ATGTCTCGCCAGTCCACCG-3′ and 5′-TTAGCTCCTGTAACTTCTCC-3′). (TIF) [file pgen.1002748.s005.tif]

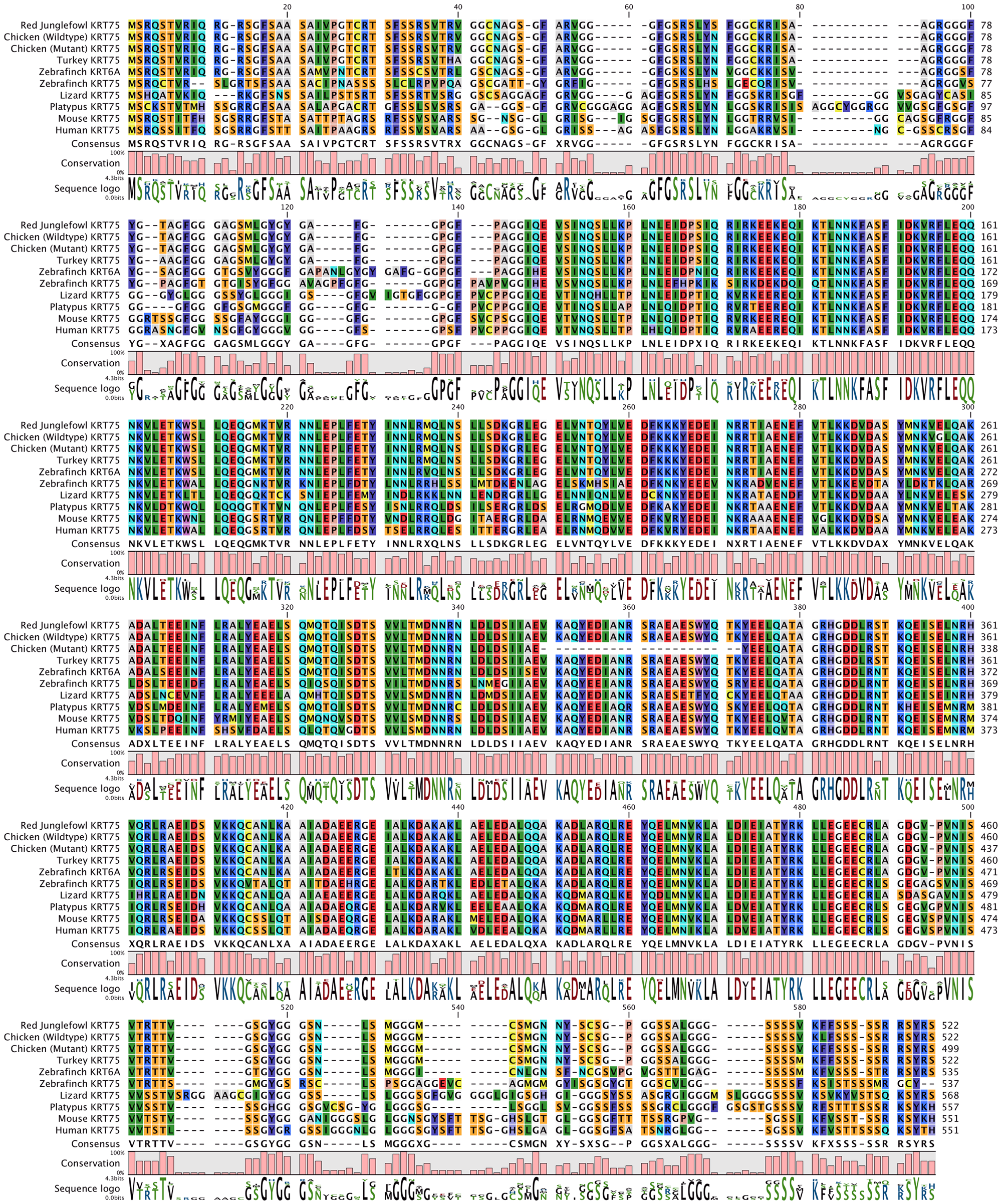

Supplement: Figure S6 — Multiple sequence alignment of amniote K75 proteins. The alignment was prepared using MUSCLE implemented in the CLC Bio 6.0 (Aarhus, Denmark). The sequences were translated from AY574985.1 (red junglefowl), XM_003205996 (turkey), XM_002194971 (zebrafinch KRT6A), XM_002194995 (zebrafinch KRT75), XM_003216971 (lizard), XM_001362788 (opossum), XM_001504398 (horse), BC137935 (mouse), and NM_004693 (human). The sequence of F allele of KRT75 has been submitted to GenBank with the accession number JQ013796. (TIF) [file pgen.1002748.s006.tif]

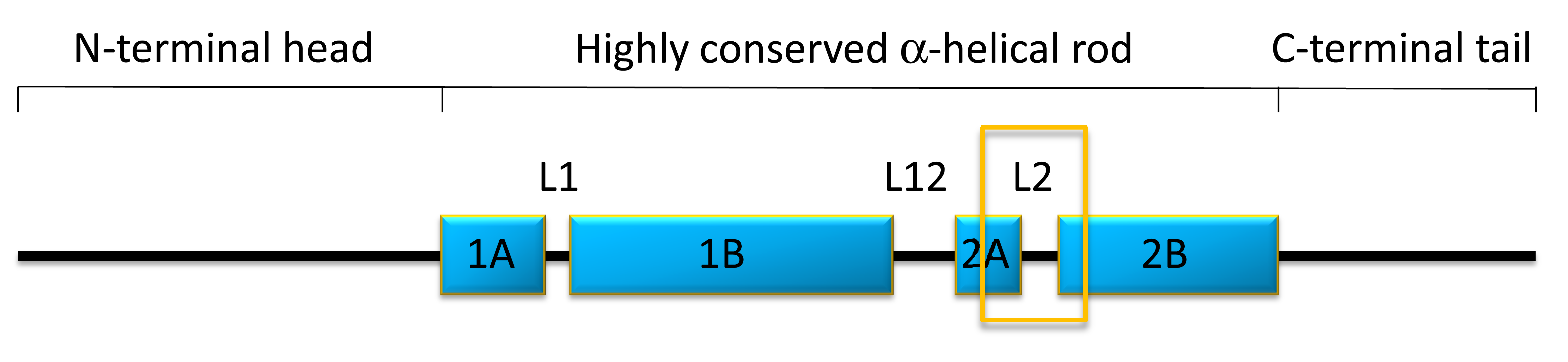

Supplement: Figure S7 — Domains of K75. The yellow-line box highlights the deletion which removes the entire part of link L2 and some parts of the coiled-coil segments of 2A and 2B. The domain information was obtained from the Human Intermediate Filament Mutation Database (www.interfil.org). (TIF) [file pgen.1002748.s007.tif]

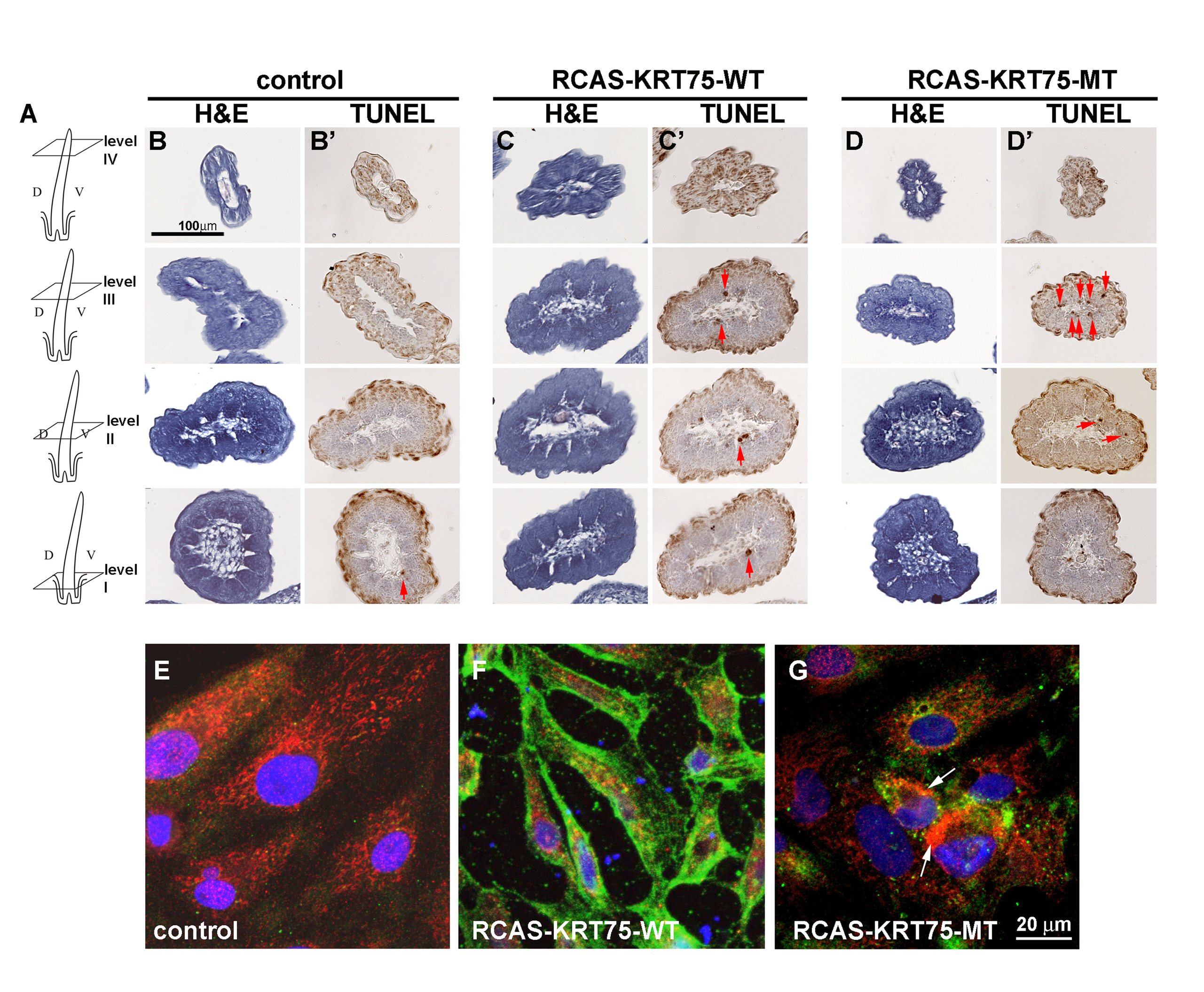

Supplement: Figure S8 — Detailed H&E and TUNEL staining at different levels among control, RCAS-KRT75-WT and RCAS-KRT75-MT mis-expression samples. (A) Diagram showing levels for section from immature (level I) to mature (level IV) regions of an E13 embryonic body feather. (B–D′) H&E and TUNEL staining at different levels. (B) and (B′) control; (C) and (C′) RCAS-KRT75-WT; (D) and (D′) RCAS-KRT75-MT. PtK2 cells stained for KRT18 (red), K75 (green) and DAPI (blue). (E) Wildtype cells; (F) RCAS-KRT75-WT; (G) RCAS-KRT75-MT. (TIF) [file pgen.1002748.s008.tif]

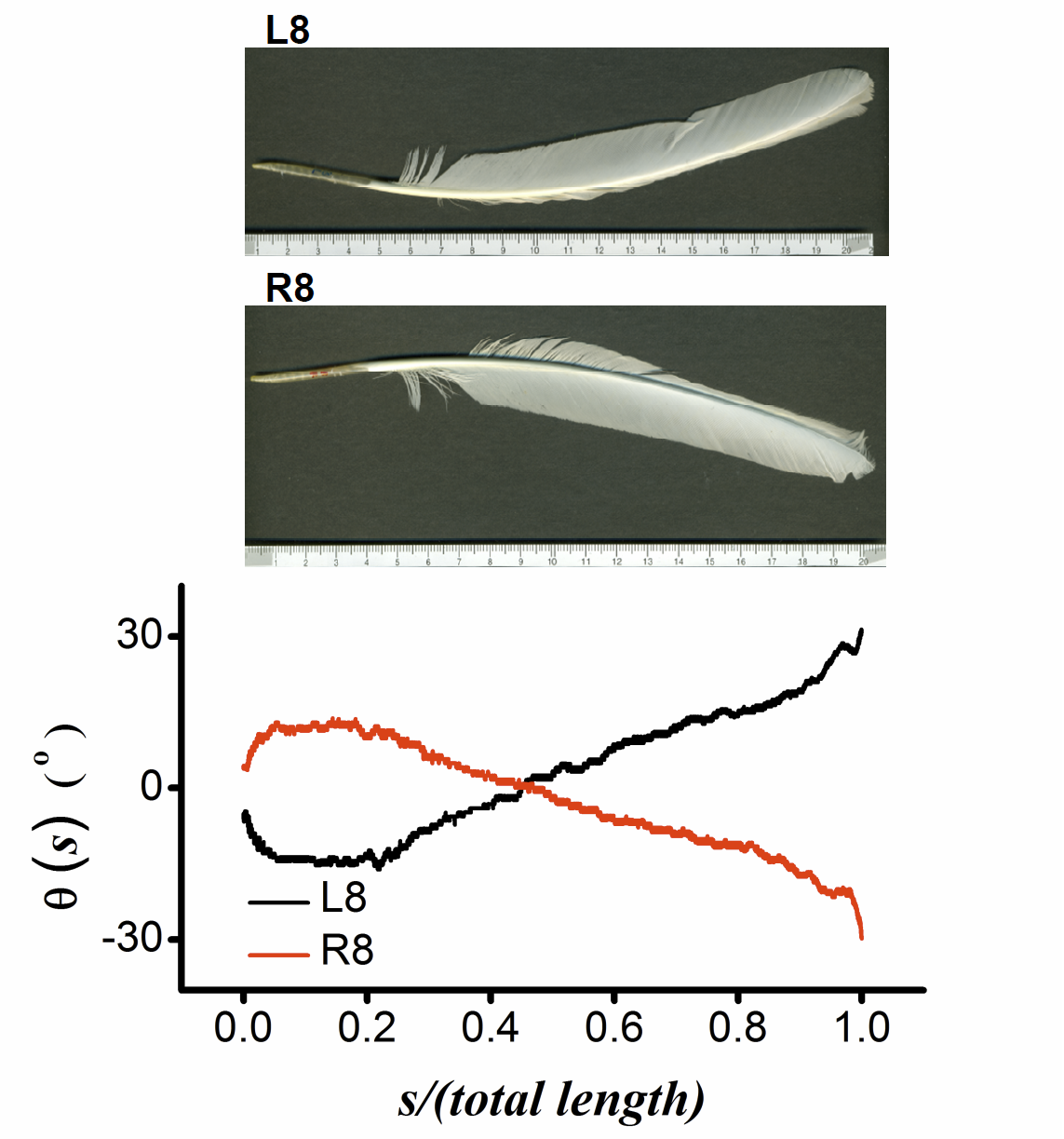

Supplement: Figure S9 — Natural bending of feathers from both sides of a chicken. (A) Images of flight feathers from opposite sides of the same normal chicken (the R8 and L8 denote the 8th feather taken from the right and left wing, respectively). (B) The trend of curves (s) shows reflective symmetry. (TIF) [file pgen.1002748.s009.tif]

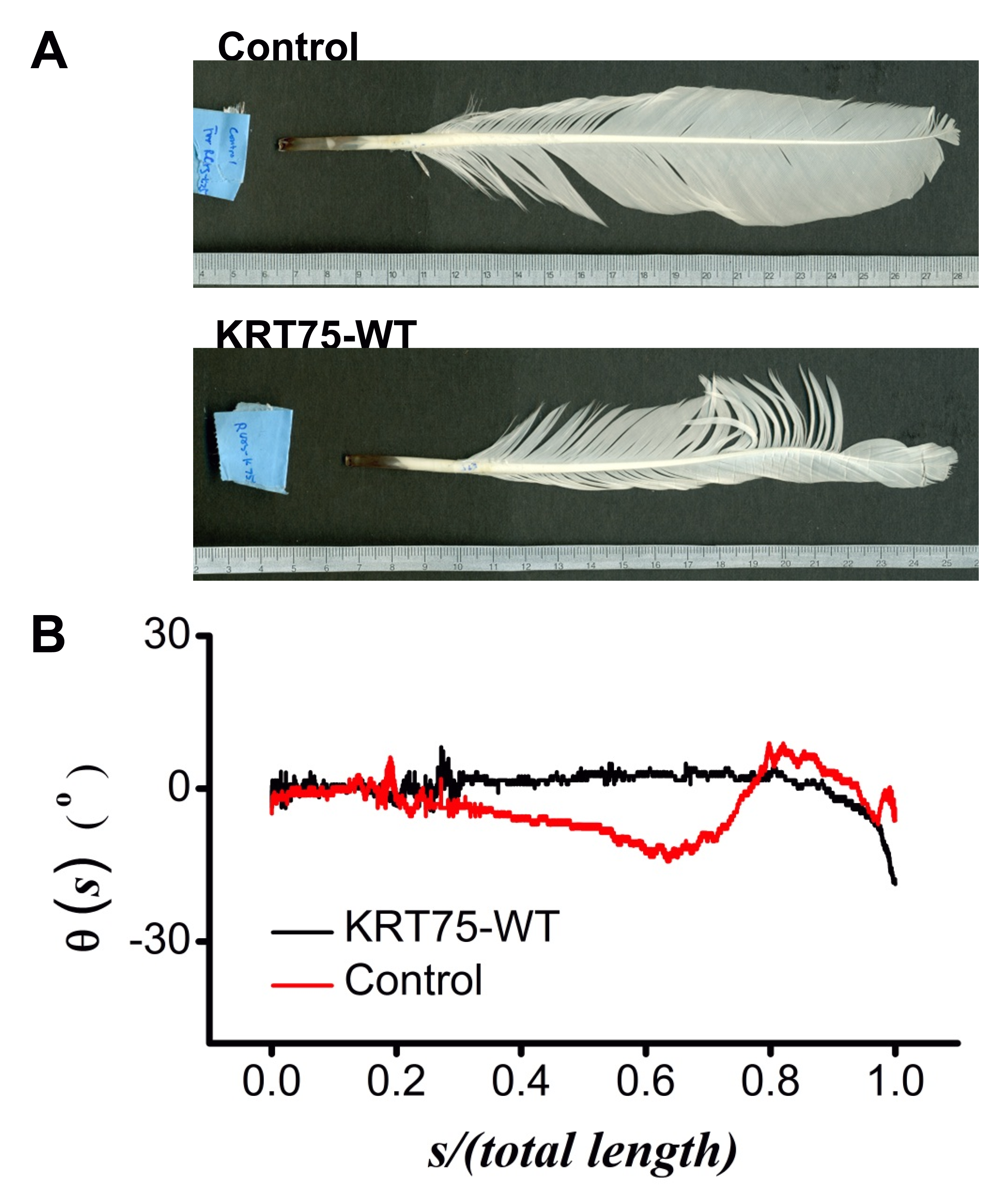

Supplement: Figure S10 — Contrast between feathers that have misexpressed GFP and overexpressed KRT75-WT. (A) Comparison of the feathers with misexpressed GFP (Control) and KRT75-WT. (B) Effects of the viral misexpression, as shown by the qualitative change of the curves of θ(s). (TIF) [file pgen.1002748.s010.tif]
