# Supplementary material for: The Chicken Frizzle Feather Is Due to an α-Keratin (KRT75) Mutation That Causes a Defective Rachis
Source: PLoS Genet. 2012 Jul 19;8(7):e1002748. doi: 10.1371/journal.pgen.1002748 (PMC3400578; doi:10.1371/journal.pgen.1002748)
Supplement: Table S1 — Primers designed to screen for the causative mutation in the candidate gene regions. (PDF) [file pgen.1002748.s011.pdf]

Table S1. Primers designed to screen for the causative mutation in the candidate gene regions.

Gene Name	Ser. No.	Start[a]	End[a]	Forward primer	Forward primer sequence	Reverse primer	Reverse primer sequence
LOC426896	A2	504379	504799	LOC426896_fwd2	AATACCCACATCCCCACACCC	LOC426896_rev2	ACAACCCAACCCCATCCCAC
LOC768882	B1	521896	522596	LOC768882_fwd1	CCTACCACAAACCTATCACC	LOC768882_rev1	CCTCCAAATCAATATCCCATTCC
LOC768882	B3	524170	524515	LOC768882_fwd3	GGGATGTTTGTAATGGGAGG	LOC768882_rev3	TGCCTTCAGCTCTCCTTATT
LOC768882	B4	524938	525568	LOC768882_fwd4	ATGGGATGGAATGCGGGGAA	LOC768882_rev4	GGACAAGGACAGGGATGCAG
LOC768882	B5	525390	525959	LOC768882_fwd5	GAGATTGAGAACGTGAAGAAG	LOC768882_rev5	AAGGAAAAGAGATGGAGTGT
LOC768882	B6	525940	526357	LOC768882_fwd6	ACACTCCATCTCTTTTCCTT	LOC768882_rev6	GTTTGCTTCCTTTCTCCTTT
LOC768882	B7	532077	532789	LOC768882_fwd7	CATTCATCCTCCTCCTCCTC	LOC768882_rev7	CTCCTCCAAATTTTTGCCCT
LOC768882	B8	535374	535838	LOC768882_fwd8	GGAGGGAGAGGAGAACAGGT	LOC768882_rev8	AGCCTGCAAAGGAAAATGAGT
LOC768882	B9	535879	536360	LOC768882_fwd9	GGTGGGCTGAGAAGTTTGGT	LOC768882_rev9	GGAAGGGAGGAGATGCTTTGT
LOC768881	C2	544762	545285	LOC768881_fwd2	ACCCTTTCGGTTTCCTCTCA	LOC768881_rev2	TTTCTTTCTCAGTCGGCCTT
LOC431299	D2	555725	556350	LOC431299_fwd2	GATTGCCCTGGATGTGGAGA	LOC431299_rev2	CCCCATTGTACACACCTCCT
LOC431299	D3	556043	556613	LOC431299_fwd3	ACATCTTGTGTTCCTCTCCCT	LOC431299_rev3	TGTTCTGCTGCTGTTTGCCT
LOC431300	E1	574421	575912	LOC431300_fwd1	GAGGAGATGGAGGGTGGTATG	LOC431300_rev1	GGACTGGAAGAAGGAGGTGA
LOC431300	E2	575893	577287	LOC431300_fwd2	TCACCTCCTTCTTCCAGTCC	LOC431300_rev2	ATCTTCTCATACTCCTGCCT
LOC431300	E4	577516	578120	LOC431300_fwd4	GCAGCCGGAAATTGAAATAGT	LOC431300_rev4	TACCTGGTCTCCTCCTCCTC
LOC768978	F1	596428	597876	LOC768978_fwd1	GTGTTTTGGAGATTTCGGCTT	LOC768978_rev1	CTCCAGGTTCTTCCCTTTCTT
LOC768978	F2	596200	596869	LOC768978_fwd2	CCTCAACTCCTGTGATCCATTCT	LOC768978_rev2	CAAATCCTATGCACCCACACT
LOC768978	F3	597856	598287	LOC768978_fwd3	AAGAAAGGGAAGAACCTGGA	LOC768978_rev3	CTGTTGGGAAAGGAGAAGAA
LOC768978	F4	598380	599795	LOC768978_fwd4	CCTTTCATGGGTTTATCTGGT	LOC768978_rev4	TCCACTTCTTCTTTGCTCTTCT
LOC768978	F5	599774	600379	LOC768978_fwd5	AGAAGAGCAAAGAAGAAGTGGA	LOC768978_rev5	GATGGAGGTTTGCAGGCAGGA
LOC768978	F6	600328	600706	LOC768978_fwd6	CCTGCACTGTTGTCCTCTCC	LOC768978_rev6	CCATCCCTTCATCCCCAGTTT
LOC431301	G1	618595	620243	LOC431301_fwd1	CATCCACCCAGAGCTCCTCA	LOC431301_rev1	GCAACGCTCCTCATTCTCCA
LOC431301	G2	618117	618874	LOC431301_fwd2	CTCCTTCACTTTCTTCCACC	LOC431301_rev2	CATCTCCCTCCTTTGTCCCA
LOC431301	G3	620216	621211	LOC431301_fwd3	AGAGAGGCTGGAGAATGAGGA	LOC431301_rev3	GTTGGAAAGCAGAGGTGGGG
LOC431301	G4	622860	623233	LOC431301_fwd4	TCAGCCCCCACCTCTGCTTT	LOC431301_rev4	CGCACCTCCAGCTCTTCCTT
LOC431301	G5	623214	624229	LOC431301_fwd5	AAGGAAGAGCTGGAGGTGCG	LOC431301_rev5	AGGGTTTTGTTGATGGAGGGT
LOC431301	G6	624207	624701	LOC431301_fwd6	AAACCCTCCATCAACAAAACC	LOC431301_rev6	CACTCAGCTCTTCACCTACC

LOC431301	G7	624523	625236	LOC431301_fwd7	TCCCAGTGTAATGTTGTCCCC	LOC431301_rev7	ACCTGCTCTCTTCTCCTTCC
LOC769040	H4	634103	635328	LOC769040_fwd4	CAGAAAAACAGCACAGGCAA	LOC769040_rev4	CTCCAGCTCCACCTTATTCA
KRT75	I1	642071	642748	KRT75_fwd1	TTTCCCCCGTTCCTGTTCTT	KRT75_rev1	CTCCTCCTGCTTTTCTTTCCT
KRT75	I2	642068	643216	KRT75_fwd2	CTCTTTCCCCCGTTCCTGTT	KRT75_rev2	CCATTTGGTCTCCAGCACTT
KRT75	I3	643120	643516	KRT75_fwd3	CGCTCTCCCTCTTCTCTTTCT	KRT75_rev3	TTTTGCTGCTGCGTGTTCATT
KRT75	I4	645094	645978	KRT75_fwd4	GGAATGGGGAATGGAATAAAAGGG	KRT75_rev4	TGCGATGATGCTGTTGAGGT
LOC408041	J1	655420	656063	LOC408041_fwd1	TCTTTCTTCTTTCCCTCCCAC	LOC408041_rev1	CCTGATCACCATTCCTTCTT
LOC408041	J2	655424	657024	LOC408041_fwd2	TCTTCTTTCCCTCCCACTCCTT	LOC408041_rev2	CGTTTTCATCCCCTGCTCCT
LOC408041	J3	657006	657726	LOC408041_fwd3	GGAGCAGGGGATGAAAACGG	LOC408041_rev3	AATGGAGGAGGCAGAGGGAG
LOC408041	J4	657707	658362	LOC408041_fwd4	CTCCCTCTGCCTCCTCCATT	LOC408041_rev4	CTCATACTGCGCCTTCACCT
LOC408041	J5	658298	659147	LOC408041_fwd5	CCATGGACAACAACCGCAAC	LOC408041_rev5	TTTCCTTCCTTCCTTCCAATCCT
LOC408042	K2	666186	666687	LOC408042_fwd2	TTCCTGTGCTGTGCTTCTTCC	LOC408042_rev2	CCTCATATTGTGCCTTCACCT
LOC408042	K3	666667	668497	LOC408042_fwd3	AGGTGAAGGCACAATATGAGGA	LOC408042_rev3	TGGGATGTGAGGCTTTTGGA
LOC426897	L1	677158	677657	LOC426897_fwd1	ACCAAGAGAAGGAGCAGATCA	LOC426897_rev1	CCCAATACAAGCCTCCAACA
LOC426897	L2	676591	677404	LOC426897_fwd2	AATCTGCTTTCTCCATTGGT	LOC426897_rev2	TTTTTGTGGGTAAGCCTTGT
LOC426897	L3	677639	679161	LOC426897_fwd3	GTTGGAGGCTTGTATTGGGA	LOC426897_rev3	CCGCCTCTCTTTTCTTCTCT
LOC426897	L4	679142	680271	LOC426897_fwd4	AGAGAAGAAAAGAGAGGCGG	LOC426897_rev4	GAAGGGGATTGTGAAGAGGA
LOC469091	M1	680018	681329	LOC469091_fwd1	GGCAAGTCGACCATGTAAAG	LOC469091_rev1	AGGAGCAGAAAGAGCAAGAA
LOC395772	N1	690505	690765	LOC395772_fwd1	TCTTTCTGTGTTTTTCCCTCCT	LOC395772_rev1	TGGTTGCTTTGCTTCTCTCT
LOC395772	N2	690581	691631	LOC395772_fwd2	TGGTGGATGAGGTTGTGGTG	LOC395772_rev2	GTTGGCTGGCGATGGAGTTG
LOC395772	N4	696860	698009	LOC395772_fwd4	ATTTCATTCTTGGTGTTGCG	LOC395772_rev4	TGAATAAAGTGGAGCTGGAGG
LOC395772	N5	703301	704000	LOC395772_fwd5	AGCTAAGTAAAGGGGGTGCAA	LOC395772_rev5	GGATAAAAGGGGAAGCGGAG

[a]Genomic positions within the linkage group chrE22C19W28_E50C23.
