# Supplementary material for: The Chicken Frizzle Feather Is Due to an α-Keratin (KRT75) Mutation That Causes a Defective Rachis
Source: PLoS Genet. 2012 Jul 19;8(7):e1002748. doi: 10.1371/journal.pgen.1002748 (PMC3400578; doi:10.1371/journal.pgen.1002748)
Supplement: Table S2 — Sequence variants within the linkage group chrE22C19W28_E50C23. (PDF) [file pgen.1002748.s012.pdf]

Table S2. Sequence variants within the linkage group chrE22C19W28_E50C23

Serial Number[a]	Position[b]	Reference genome[c]	Frizzle[d]	Variant type[e]	Coding or noncoding[e]
A2	504442	T	A	SNP	Noncoding
A2	504547	A	G	SNP	Noncoding
B1	522166	T	C	Synonymous SNP	Coding
B1	522488	C	T	SNP	Noncoding
B3	524198	T	C	SNP	Noncoding
B3	524268	G	A	SNP	Noncoding
B3	524289	T	C	SNP	Noncoding
B3	524307-524310	AAAC	-	SNP	Noncoding
B3	524491	T	G	SNP	Noncoding
B4	525048	A	G	Synonymous SNP	Coding
B4	525160	A	T	Nonsynonymous SNP (S to C)[f]	Coding
B4	525220	C	A	SNP	Noncoding
B4	525255	A	C	SNP	Noncoding
B4	525528	C	A	SNP	Noncoding
B5	525797	G	A	SNP	Noncoding
B6	525808	G	T	SNP	Noncoding
B6	525865	T	C	SNP	Noncoding
B6	526043	G	A	SNP	Noncoding
B6	526089	G	A	SNP	Noncoding
B6	526231	A	C	SNP	Noncoding
B6	526233	A	G	SNP	Noncoding
B6	526254	C	G	SNP	Noncoding
B6	526257	C	G	SNP	Noncoding
B7	532697	A	G	SNP	Noncoding
B7	532707	A	C	SNP	Noncoding
B8	535589	T	C	SNP	Noncoding

B8	535831	-	T	Insertion	Noncoding
C2	545226-545228	CAC	-	Frame-shift deletion[g]	Coding
D2	556094	C	T	SNP	Noncoding
D3	556094	C	T	SNP	Noncoding
E1	574608	A	G	SNP	Noncoding
E1	574610	G	C	SNP	Noncoding
E1	574737	G	A	SNP	Noncoding
E1	574749	T	A	SNP	Noncoding
E1	575274	T	C	SNP	Noncoding
E1	575663	C	G	SNP	Noncoding
E2	576436	G	A	SNP	Noncoding
E2	576579	A	C	SNP	Noncoding
E2	576883	C	A	SNP	Noncoding
E2	576893	G	A	SNP	Noncoding
E4	577719	A	C	SNP	Noncoding
E4	577720	A	C	SNP	Noncoding
E4	577721	G	C	SNP	Noncoding
F3	597969	A	G	SNP	Noncoding
F4	598543	C	T	SNP	Noncoding
F4	598587	G	C	SNP	Noncoding
F4	598589	A	G	SNP	Noncoding
F4	598636	A	G	SNP	Noncoding
G1	618713	G	T	SNP	Noncoding
G1	618928	A	G	SNP	Noncoding
G1	618934	G	A	SNP	Noncoding
G2	618165	T	C	SNP	Noncoding
G2	618713	G	T	SNP	Noncoding
G3	620444	A	G	SNP	Noncoding

G3	620478	T	C	SNP	Noncoding
G3	620581	T	C	SNP	Noncoding
G3	620673	C	G	SNP	Noncoding
G3	620838	A	T	SNP	Noncoding
G3	620858	T	G	SNP	Noncoding
G3	620859	T	C	SNP	Noncoding
G3	620890	A	T	SNP	Noncoding
G3	620978	G	C	SNP	Noncoding
G3	621041	G	T	SNP	Noncoding
G4	622859	A	T	SNP	Noncoding
G4	623234-623319	…	-	86-bp deletion	Noncoding
G5	623387	A	G	SNP	Noncoding
G5	623517	T	C	SNP	Noncoding
G5	623609	C	T	SNP	Noncoding
G5	623610	A	G	SNP	Noncoding
G5	623623	A	G	SNP	Noncoding
G5	623626	A	G	SNP	Noncoding
G7	624694	G	C	SNP	Noncoding
G7	625199	G	A	SNP	Noncoding
H4	634622	A	G	SNP	Noncoding
H4	634654	T	A	SNP	Noncoding
H4	634707	C	T	SNP	Noncoding
H4	634708	C	G	SNP	Noncoding
H4	634945	G	T	SNP	Noncoding
H4	634946	C	T	SNP	Noncoding
H4	634963	C	T	SNP	Noncoding
H4	635102	T	C	SNP	Noncoding
H4	635131	G	A	SNP	Noncoding

H4	635209	T	C	SNP	Noncoding
I1	642283	C	T	Synonymous SNP	Coding
I1	642514	T	C	Synonymous SNP	Coding
I2	642514	T	C	Synonymous SNP	Coding
I2	642821	C	T	SNP	Noncoding
I2	642876	C	A	SNP	Noncoding
I2	643029	G	T	SNP	Noncoding
I2	643088	C	T	SNP	Noncoding
I2	643149	T	-	Deletion	Noncoding
I3	643468	T	-	Deletion	Noncoding
I4	645216	G	A	SNP	Noncoding
I4	645239	T	C	SNP	Noncoding
I4	645251	G	A	SNP	Noncoding
I4	645318	G	A	SNP	Noncoding
I4	645390	A	T	Nonsynonymous SNP (K to N)[g]	Coding
I4	645493	T	C	SNP	Noncoding
I4	645613	G	A	SNP	Noncoding
I4	645627	T	C	SNP	Noncoding
I4	645838	T	C	SNP	Noncoding
I4	645880	T	C	SNP	Noncoding
J1	646203	G	A	SNP	Noncoding
J1	646267	G	A	SNP	Noncoding
J1	646363	G	A	SNP	Noncoding
J1	646677	A	G	SNP	Noncoding
J1	646695	T	C	SNP	Noncoding
J1	646720	T	C	SNP	Noncoding
J1	646747	A	G	SNP	Noncoding
J1	646848	G	T	SNP	Noncoding

J1	646993	C	T	SNP	Noncoding
J2	655694	G	A	Nonsynonymous SNP (S to N)[h]	Coding
J2	656017	A	G	SNP	Noncoding
J2	656151	T	C	SNP	Noncoding
J2	656235	T	C	SNP	Noncoding
J2	656278	A	G	SNP	Noncoding
J2	656288	A	C	SNP	Noncoding
J2	656370	T	A	SNP	Noncoding
J2	656373-656374	TT	-	Deletion	Noncoding
J3	657221	T	C	SNP	Noncoding
J3	657248	C	T	SNP	Noncoding
J3	657257	T	G	SNP	Noncoding
J3	657290	G	A	SNP	Noncoding
J3	657297	G	A	SNP	Noncoding
J3	657553	C	T	SNP	Noncoding
J4	657864	T	C	SNP	Noncoding
J4	657909	A	C	SNP	Noncoding
J4	657976	T	C	Synonymous SNP	Coding
J4	657982	T	C	Synonymous SNP	Coding
J4	658290	A	G	Synonymous SNP	Coding
J5	658380	T	C	Synonymous SNP	Coding
**J5**	**658389-658472**	**…**	**-**	**84-dp deletion**[i]	**Coding & noncoding**
J5	658640	A	G	SNP	Noncoding
J5	658653	G	A	SNP	Noncoding
J5	658733	C	A	SNP	Noncoding
J5	658826	G	A	SNP	Noncoding
J5	658830	C	T	SNP	Noncoding
J5	658940	G	C	SNP	Noncoding

J5	658959	T	C	Synonymous SNP	Coding
J5	659067	T	C	Synonymous SNP	Coding
J5	659088	G	A	SNP	Noncoding
K3	667772	T	C	Synonymous SNP	Coding
K3	668083	C	G	SNP	Noncoding
K3	668149	T	A	Synonymous SNP	Coding
K3	668253	C	T	Synonymous SNP	Coding
L1	677360	C	T	SNP	Noncoding
L1	677442	T	C	SNP	Noncoding
L1	677466	C	T	SNP	Noncoding
L1	677500	G	A	SNP	Noncoding
L1	677518	C	G	SNP	Noncoding
L2	676657	T	C	SNP	Noncoding
L2	676821	G	A	SNP	Noncoding
L2	677048	T	C	SNP	Noncoding
L2	677082	G	A	SNP	Noncoding
L2	677275	A	G	SNP	Noncoding
L2	677360	C	T	SNP	Noncoding
L3	677915	G	T	SNP	Noncoding
L3	678280	A	C	SNP	Noncoding
L3	678484	T	C	SNP	Noncoding
L4	679752	G	A	SNP	Noncoding
L4	679897	T	C	SNP	Noncoding
L4	679976	C	T	SNP	Noncoding
L4	679989	A	G	SNP	Noncoding
L4	680120	A	G	SNP	Noncoding
M1	680120	A	G	SNP	Noncoding
N1	688128	G	T	Synonymous SNP	Coding

N1	688161	G	T	Synonymous SNP	Coding
N1	688173	A	G	Synonymous SNP	Coding
N2	690741	G	A	SNP	Noncoding
N2	690744	G	A	SNP	Noncoding
N2	690880	T	A	SNP	Noncoding
N2	690939	A	G	SNP	Noncoding
N2	690967	G	A	SNP	Noncoding
N2	691110	A	G	SNP	Noncoding
N2	691125	A	G	SNP	Noncoding
N2	691133	C	T	SNP	Noncoding
N2	691162	C	A	SNP	Noncoding
N2	691163	C	A	SNP	Noncoding
N2	691259	G	A	SNP	Noncoding
N4	697062	A	G	Synonymous SNP	Coding
N4	697179	G	A	Synonymous SNP	Coding
N4	697298	G	A	SNP	Noncoding
N4	697306	G	C	SNP	Noncoding
N4	697568	A	T	SNP	Noncoding
N4	697754	G	A	SNP	Noncoding
N4	697758	C	T	SNP	Noncoding
N5	703807	A	G	Synonymous SNP	Coding
N5	703879	G	A	Synonymous SNP	Coding

[a]Primers used to obtain the sequences from which the variants were discovered are listed in Table S1.
[b]Genomic positions within the linkage group chrE22C19W28_E50C23.
[c]Polymorphisms in the chicken reference genome (WUGSC 2.1/galGal3) [1]. Sequence longer than 80 bp are showed as "…".
[d]Variants detected in the frizzle chicken compared to the chicken reference genome.
[e]Variants are annotated using the Ensembl genome database (http://www.ensembl.org/).

[f]The amino acid substituion is predicted to affect protein functions by the bioinformatics tool SIFT [2,3,4], but the confidence in this prediction is low because the sequences used were not diverse enough. The effect of this SNP has not been experimentally studied.
[g]The same variants were also found in the white leghorn chicken with normal feathers. The effect of these SNPs have not been experimentally studied.
[h]The same variant has also been reported (GenBank accession number AY574985.1).
[i]This sequence has been submitted to GenBank with the accession number JQ013796.

Reference:

1. Hillier LW, Miller W, Birney E, Warren W, Hardison RC, et al. (2004) Sequence and comparative analysis of the chicken genome provide unique perspectives on vertebrate evolution. Nature 432: 695-716.
2. Ng PC, Henikoff S (2001) Predicting deleterious amino acid substitutions. Genome Res 11: 863-874.
3. Ng PC, Henikoff S (2003) SIFT: Predicting amino acid changes that affect protein function. Nucleic Acids Res 31: 3812-3814.
4. Kumar P, Henikoff S, Ng PC (2009) Predicting the effects of coding non-synonymous variants on protein function using the SIFT algorithm. Nature Protocols 4: 1073-1082.
